# Detection of SARS-CoV-2 in Air and on Surfaces in Rooms of Infected Nursing Home Residents

**DOI:** 10.1093/annweh/wxac056

**Published:** 2022-09-07

**Authors:** Kimberly J Linde, Inge M Wouters, Jan A J W Kluytmans, Marjolein F Q Kluytmans-van den Bergh, Suzan D Pas, Corine H GeurtsvanKessel, Marion P G Koopmans, Melanie Meier, Patrick Meijer, Ceder R Raben, Jack Spithoven, Monique H G Tersteeg-Zijderveld, Dick J J Heederik, Wietske Dohmen, COCON Consortium

**Affiliations:** Institute for Risk Assessment Sciences, Utrecht University, Utrecht, The Netherlands; Institute for Risk Assessment Sciences, Utrecht University, Utrecht, The Netherlands; Julius Center for Health Sciences and Primary Care, University Medical Center Utrecht, Utrecht University, Utrecht, The Netherlands; Julius Center for Health Sciences and Primary Care, University Medical Center Utrecht, Utrecht University, Utrecht, The Netherlands; Department of Infection Control, Amphia Hospital, Breda, The Netherlands; Microvida Location Amphia/Bravis, Breda/Roosendaal, The Netherlands; Department of ViroScience, Erasmus MC, Rotterdam, The Netherlands; Department of ViroScience, Erasmus MC, Rotterdam, The Netherlands; Mijzo, Waalwijk, The Netherlands; Institute for Risk Assessment Sciences, Utrecht University, Utrecht, The Netherlands; Institute for Risk Assessment Sciences, Utrecht University, Utrecht, The Netherlands; Institute for Risk Assessment Sciences, Utrecht University, Utrecht, The Netherlands; Institute for Risk Assessment Sciences, Utrecht University, Utrecht, The Netherlands; Institute for Risk Assessment Sciences, Utrecht University, Utrecht, The Netherlands; Institute for Risk Assessment Sciences, Utrecht University, Utrecht, The Netherlands

**Keywords:** air levels, nursing home, SARS-CoV-2, surface

## Abstract

There is an ongoing debate on airborne transmission of Severe Acute Respiratory Syndrome Coronavirus 2 (SARS-CoV-2) as a risk factor for infection. In this study, the level of SARS-CoV-2 in air and on surfaces of SARS-CoV-2 infected nursing home residents was assessed to gain insight in potential transmission routes. During outbreaks, air samples were collected using three different active and one passive air sampling technique in rooms of infected patients. Oropharyngeal swabs (OPS) of the residents and dry surface swabs were collected. Additionally, longitudinal passive air samples were collected during a period of 4 months in common areas of the wards. Presence of SARS-CoV-2 RNA was determined using RT-qPCR, targeting the RdRp- and E-genes. OPS, samples of two active air samplers and surface swabs with Ct-value ≤35 were tested for the presence of infectious virus by cell culture. In total, 360 air and 319 surface samples from patient rooms and common areas were collected. In rooms of 10 residents with detected SARS-CoV-2 RNA in OPS, SARS-CoV-2 RNA was detected in 93 of 184 collected environmental samples (50.5%) (lowest Ct 29.5), substantially more than in the rooms of residents with negative OPS on the day of environmental sampling (*n* = 2) (3.6%). SARS-CoV-2 RNA was most frequently present in the larger particle size fractions [>4 μm 60% (6/10); 1–4 μm 50% (5/10); <1 μm 20% (2/10)] (Fischer exact test *P* = 0.076). The highest proportion of RNA-positive air samples on room level was found with a filtration-based sampler 80% (8/10) and the cyclone-based sampler 70% (7/10), and impingement-based sampler 50% (5/10). SARS-CoV-2 RNA was detected in 10 out of 12 (83%) passive air samples in patient rooms. Both high-touch and low-touch surfaces contained SARS-CoV-2 genome in rooms of residents with positive OPS [high 38% (21/55); low 50% (22/44)]. In one active air sample, infectious virus *in vitro* was detected. In conclusion, SARS-CoV-2 is frequently detected in air and on surfaces in the immediate surroundings of room-isolated COVID-19 patients, providing evidence of environmental contamination. The environmental contamination of SARS-CoV-2 and infectious aerosols confirm the potential for transmission via air up to several meters.

**What’s Important About This Paper?**

This study, conducted in a nursing home, provides insights into the extent of SARS-CoV-2 presence in air and on surfaces around patients, and the infectivity of aerosols. These insights can contribute to the discussion on potential airborne transmission of SARS-CoV-2 and facilitates effective design of prevention strategies such as use of facemasks, respirators, and ventilation.

## Introduction

The ongoing pandemic, caused by Severe Acute Respiratory Syndrome Coronavirus 2 (SARS-CoV-2), continues to constitute a public health emergency of international concern. There is consensus on the role of direct contact transmission and airborne transmission at short distances (up to 3 m) through large droplets caused by e.g. couhing and sneezing. There is an ongoing debate on transmission through fomites and airborne transmission through smaller droplets caused by e.g. speaking and breathing at larger distances (up to several meters) as a risk factor for subsequent infection (Lednicky *et al.*, 2020; Sikkema *et al.*, 2020). The relative importance of this mode of transmission as driver of the pandemic is unknown. Several modes of transmission through the environment as a possible risk of infection of SARS-CoV-2 is considered important for groups at high risk (Cherrie *et al.*, 2021).

Previous studies investigating SARS-CoV-2 air concentrations in healthcare facilities showed contradictory results. In a limited number of studies in hospital settings, SARS-CoV-2 has been detected in air in proximity (2–5 m) of COVID-19 patients (Chia *et al.*, 2020; Guo *et al.*, 2020; Lednicky *et al.*, 2020; Liu *et al.*, 2020; Santarpia *et al.*, 2020; Conway-Morris *et al.*, 2021). Other studies did not find evidence of SARS-CoV-2 in air (Faridi *et al.*, 2020; Masoumbeigi *et al.*, 2020; Lane *et al.*, 2021; Vosoughi *et al.*, 2021). However, the comparability of studies is limited due to differences in sampling methods, sampling duration, and distance to infected persons. Oropharyngeal swabs were not consistently collected from infected persons for confirmation of infection and the actual level SARS-CoV-2 shedding in addition to the collection of air samples. Infectiousness of SARS-CoV-2 detected in air was not investigated in most studies (Chia *et al.*, 2020; Guo *et al.*, 2020; Liu *et al.*, 2020; Conway-Morris *et al.*, 2021) or could not be shown (Döhla *et al.*, 2022; Nannu Shankar *et al.*, 2022). Infectivity and amount of shed virus have been reported to rapidly decline during the first week after illness onset (van Beek *et al.*, 2021; van Kampen *et al.*, 2021). As viral RNA can persist and be shed for prolonged periods of time without being infectious, it is important to investigate the viability of virus in air to understand airborne transmission routes of the virus. Therefore, to successfully investigate modes of transmission of SARS-CoV-2, it seems crucial to investigate SARS-CoV-2 air concentrations in the first days following infection.

After the first pandemic wave in The Netherlands, nursing homes had introduced enhanced surveillance screening for SARS-CoV-2, which led to the identification of new infections at an early stage (National Institute for Public Health and the Environment, 2021). To investigate potential airborne transmission routes from SARS-CoV-2 infected patients to their immediate surroundings, we measured SARS-CoV-2 in air and on surfaces in Dutch nursing home residencies as well as in rooms of SARS-CoV-2 isolated infected nursing home residents.

## Methods and materials

**Figure 1.**
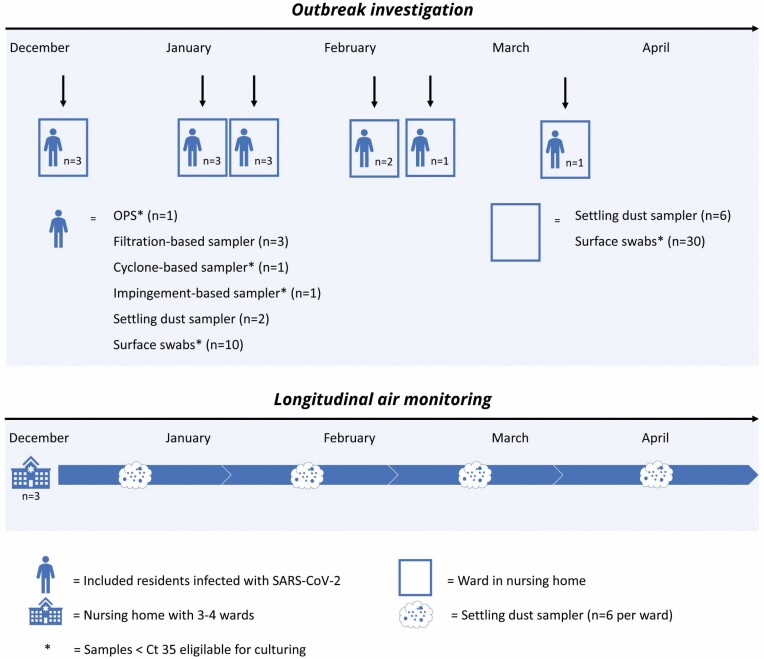
Design of the study. The study consisted of an outbreak investigation which was complemented by longitudinal air monitoring

The study consisted of two arms: a series of environmental investigations during outbreaks and longitudinal air monitoring (Fig. 1). Weekly SARS-CoV-2 infections were registered and notified in 28 nursing homes from Mijzo Care organisation in Noord-Brabant in the Netherlands. In case of two or more confirmed SARS-CoV-2 infections in residents within the same ward, an outbreak investigation was initiated, consisting of extensive environmental sample collection and SARS-CoV-2 testing of persons. In a subsample of 3 of the 28 nursing homes, longitudinal monitoring took place in the direct living environment. The study protocol was evaluated by the Medical Research Ethics Committee of University Medical Centre Utrecht. As the study did not fall within the scope of the Dutch Act on Medical Research Involving Human Subjects no further medical ethical approval was required (METC protocol no. 20-277/C). The study was conducted in agreement with the European legislation on handling privacy-sensitive data.

### Outbreak investigation

Residents of the nursing homes were tested for possible SARS-CoV-2 infection in case they experienced COVID-19-related symptoms. When one or more residents in a ward tested positive for SARS-CoV-2 infection, all other ward residents were screened for SARS-CoV-2 infection during surveillance rounds. Residents who tested positive for SARS-CoV-2 RNA were eligible for inclusion in the outbreak investigation within 8 days since the onset of symptoms or within 8 days since the first positive surveillance test result. Only patients in isolation from somatic wards were included. Oral informed consent was obtained from patients and/or from an authorized legal representative or family member.

#### Collection of air samples

Air samples were collected at three locations in the patient’s room: (i) near the head of the patients within approximately 0.5 m of the patient, (ii) near the feet of bedridden patients approximately 1.5 m from the head or approximately 1.5 m from mobile patients sitting in a chair, and (iii) near the location often used by healthcare workers more than 2 m away from the patient such as the sink, all positioned at 1.5 m height. In every patient room, 6-hr inhalable dust samples were taken using a filtration-based technique at all three locations [Conical Inhalable dust Sampler (CIS), JS Holdings, UK]. In addition, one 6-hr two-stage cyclone-based sample with filter back-up was positioned near the feet of the patient when bedridden or at 1.5 m from the chair of the patient (NIOSH BC 251, kindly provided by Dr William G Lindsley, NIOSH CDC, Morgantown, USA), as well as a 1-hr impingement-based sampler positioned in proximity of the head of the patient (5 ml BioSampler, SKC, UK) [see Supplement Fig. S1 (available at *Annals of Occupational Hygiene* online)]. During the 6-hr sample collection, mobile patients were allowed to move in the room. During the 1-hr impingement-based sample collection, they were asked to stay seated in their chair. The filtration-based sampler was equipped with a 37 mm diameter 2.0 μm pore-size Teflon filter (Pall incorporated, Ann Arbor, USA). The two-stage cyclone-based sampler allowed size-selective sampling and was equipped with two conical tubes (of 15 ml and 1.5 ml) which sample respectively particulates of 1–4 μm and >4 μm, and a back-up Teflon filter (37 mm diameter 2.0 μm pore-size Pall incorporated, Ann Arbor, USA) for particulates of <1 μm when operated at a flow of 3.5 l min^‐1^. The 15 ml and 1.5 ml conical tubes were filled with virus transport medium 1 (VTM-1; Erasmus Medical Center (EMC), Rotterdam, The Netherlands) during sampling and Opti-MeM (Gibco, UK) was added immediately after collection [see Supplementary methods (available at *Annals of Occupational Hygiene* online) for more details and composition of media]. Adding VTM-1 is a modification of the standard operating procedure for this sampler with the aim to enhance the culturability of the virus. The impingement-based sampler contained VTM-1 during sampling, and after completion of sampling, Opti-MeM was added as well.

Airborne settling dust was sampled using Electrostatic Dust Collectors (EDCs) (Noss *et al.*, 2008), which were placed in each included patient room and the corresponding hallway, common living room, and nurse office of the ward. EDCs were placed in holders pinned to the ceiling in the middle of the space, approximately 30 cm underneath the ceiling or on top of a cabinet. EDCs were collected after 2–4 weeks of sampling, dependent on the timing of extensive cleaning of the room.

#### Collection of surface samples

High- and low-touch surface samples were collected using dry surface swabs (Medical Wire Dry Swabs, MW730, Corsham, UK) as described previously (WHO and World Health Organization, 2020; de Rooij *et al.*, 2021). A total of 10 samples were taken in each patient room, and in the corresponding hallway, common living room, and nurse office of the ward. Disposable plastic grids of 10 cm^2^ were used to standardize collection of surface swabs. Swabs were placed in viral transport medium 2 (VTM-2; Erasmus Medical Center (EMC), Rotterdam, The Netherlands) directly after collection [see Supplementary methods (available at *Annals of Occupational Hygiene* online) for the composition of media].

Field blank samples were collected every other outbreak sampling day for each air sampling technique and every outbreak sampling day for surface swab sampling. See Supplementary methods (available at *Annals of Occupational Hygiene* online) for details on sample collection and laboratory methods.

#### Patient characteristics

Patient characteristics were obtained: gender, year of birth, date of symptom onset, symptoms, date of SARS-CoV-2 test, SARS-CoV-2 test results, COVID-19 treatment such as oxygen therapy and mobility. An affirmative oropharyngeal swab (OPS) (Medical Wire Dry Swabs, 111598, Milan, Italy) was collected during the outbreak investigation and stored in a tube containing VTM-2.

### Longitudinal air monitoring

Three of the 28 nursing homes with at least three wards (somatic and/or geriatric) were selected for longitudinal air monitoring. In each selected nursing home, settling dust samples were collected repeatedly in three to four wards from December 2020 until May 2021. Per ward, six EDCs were placed in hallways, living rooms, and nurse offices and renewed every four weeks for a period of four months. Incidence of SARS-CoV-2 infections in patients and staff members at the included wards was obtained in weekly reports.

### Laboratory analysis

All samples, except settling dust samples, were stored and sent to the laboratory refrigerated at 4°C directly after collection. At the laboratory, samples were stored at 4°C until further processing within 24-hr under biosafety laboratory (BSL)-2+ conditions (Duane, 2013). Filters were removed from the filter holder and transferred to a tube containing VTM-1. These and all other outbreak investigation samples were subsequently vortexed. Settling dust samples were transferred to tubes containing VTM-2 and tamped down, vortexed, and soaked repeatedly for several minutes. For RT-qPCR analysis, an aliquot of VTM was mixed in a 1:1 dilution with MagNA Pure 96 External Lysis Buffer for each sample (Roche Diagnostics, Almere the Netherlands). The remaining VTM from cyclone-based samples, impingement-based samples, surface swabs, and OPS were stored for culturing. All samples were stored frozen at ‐80°C until further processing. More details are described in Supplementary methods (available at *Annals of Occupational Hygiene* online).

#### Real-time semi-quantitative reverse transcriptase polymerase chain reaction

VTM-lysis buffer samples were tested for the presence of SARS-CoV-2 RNA using a SARS-CoV-2 RNA RT-qPCR, targeting the E-gene and CoV-2 RdRp-gene of SARS-CoV-2 using the cobas 6800/8800 Systems (Roche Diagnostics) (Stohr *et al.*, 2020). If both E-gene and RdRp-gene were detected with Cobas RT-qPCR, samples were classified as positive. In case of a discrepant result, i.e. only one of the two genes was detected, an in-house RT-qPCR assay was conducted for confirmation (Kluytmans-Van Den Bergh *et al.*, 2020). In the case of detection of SARS-CoV-2 RNA, the sample was classified as positive and in the case of inconclusive or nondetection with in-house RT-qPCR, the final result was classified as inconclusive. Samples with nondetection of both genes were classified as negative.

#### Virus culture

OPS, cyclone-based samples, impingement-based samples, and surface swabs tested positive by RT-qPCR with RdRp Ct ≤35 were tested for infectious SARS-CoV-2, as described previously (van Kampen *et al.*, 2021). Virus culture was performed in 24-wells plates seeded with Vero cells, clone 118. Samples were added to the wells, centrifuged, and inoculum was discarded. Virus culture medium was added, and samples were cultured at 37°C and 5% CO_2_ for seven days. If a virus-induced cytopathic effect (CPE) was observed, immunofluorescent detection of SARS-CoV-2 nucleocapsid protein was performed to confirm the presence of SARS-CoV-2.

### SARS-CoV-2 Whole Genomen Sequencing (WGS)

In samples with RT-PCR RdRp Ct-values <31 whole genome sequencing was performed on the primary clinical specimen by Microvida to determine the SARS-CoV-2 variant. Genomes with >90% genome coverage were included. For more details see Supplementary Methods (available at *Annals of Occupational Hygiene* online).

### Data analysis

Data entry was carried out in Microsoft Access Version 16 2012. Descriptive statistics were obtained by R studio Version 1.4.1106 2021. Active air sample techniques were compared on room level. If one or more of the filtration-based samples in a room were positive, the outcome on room level was classified as positive. The same applied for the CDC-NIOSH cyclone-based samples on room level. Fisher’s exact test was used to compare the proportion of positive samples in association with particle size fractions, distance, and location, and to compare air sampling techniques. Agreement between outcomes of filtration-based and cyclone-based samples collected at the same location was investigated through Cohen’s Kappa test statistics. A threshold of 0.05 was used for the *P*-value for statistical significance.

## Results

A total of 679 environmental samples were collected from five nursing home wards, including 101 air samples and 122 surface samples from the patient rooms and 259 air samples and 197 surface samples from common areas. In total, 13 patients were included for environmental sample collection during outbreak investigations. One patient withdrew from the study during sampling. Of the remaining 12 patients, 2 tested negative, and 10 tested positive in affirmative OPS collected on the day of environmental sample collection (Table 1). From one of the two patients with negative OPS only surface swabs were collected because the patient retracted participation to the study after surface swab sampling. For air samples, RdRp Ct-values ranged from 29.5 to 37.2 and from 30.2 to 37.8 in surface swab and from 19.8 to 34.7 in OPS. All field and laboratory blanks tested negative for viral SARS-CoV-2 RNA.

**Table 1.** SARS-CoV-2 PCR in environmental samples in the surrounding of isolated patients and common areas in nursing homes

Outbreak	Location	Patient characteristics			Environmental samples						
		Day of illness	Mobility	Oropharyngeal swab (RdRp CT)	Active air sampling					Passive (air) sampling	
					CIS-inhalable dust	CDC-NIOSH bioaerosol sampler-cyclone-based			SKC Bio-sampler -Impinger	EDC-settling dust	Surface swabs
					PTFE filter	PTFE filter (<1 μm)	Microcentrifuge tube 1.5 ml (1–4 μm)	Centrifuge tube 15 ml (>4 μm)			
*A*	Patient room 10	4	No	**19.8** ^**C**^	**3**/0/0	**1**/0/0	**1**/0/0	**1**/0/0 ^C^	**1**/0/0	NO	**4**/1/5
	Patient room 11^x^	6	Yes	Neg	NO	NO	NO	NO	NO	0/1/0	**1**/0/9
	Patient room 12	7	No^OX^	**29.6**	**2**/1/0	0/0/1	**1**/0/0	0/1/0	0/0/1	NO	**3**/0/6
	Common areas	–	–	–	–	–	–	–	–	**2**/1/3	0/0/20*
*B*_1_	Patient room 13	1	No^OX^	**27.6** ^**C**^	**3**/0/0	0/0/1	**1**/0/0	**1**/0/0	**1**/0/0	**1**/0/0	**9**/0/1
	Patient room 14	AS	Yes	Neg	0/0/3	0/0/1	0/0/1	0/0/1	0/0/1	0/1/1	0/0/10
	Patient room 15	Unclear	Yes	**29.7**	0/0/3	0/0/1	0/0/1	0/0/1	**1**/0/0	**1**/0/0	**5**/1/4
	Common areas	–	–	–	–	–	–	–	–	**3**/0/2	0/0/30
*B*_2_	Patient room 16	AS	Yes	**32.8**	**2**/0/1	0/0/1	0/0/1	**1**/0/0	0/0/1	**2**/0/0	**4**/0/6
	Patient room 17	AS	Yes	**19.9** ^**C**^	**3**/0/0	**1**/0/0	**1**/0/0	**1**/0/0	**1**/0/0	**2**/0/0	**5**/1/4
	Patient room 18	1	Yes	**33.6**	**2**/0/1	0/0/1	0/0/1	**1**/0/0	0/0/1	**1**/0/1	**4**/0/6
	Common areas	–	–	–	–	–	–	–	–	**3**/0/2	**4**/0/26
C**	Patient room 19^x^	5	Yes	NO	0/0/3	0/0/1^●^	0/0/1^●^	0/0/1^●^	NO	0/0/2	NO
	Common areas	–	–	–	–	–	–	–	–	NO	0/0/30
	Patient room 20	5	Yes	**34.7**	**3**/0/0	0/0/1	**1**/0/0	**1**/0/0	**1**/0/0	**2**/0/0	**12**/0/1^***^
	Common areas	–	–	–	–	–	–	–	–	NO	**1**/0/26
*D* *E*	Patient room 22	3	No^OX^	**30.8**	**1**/0/2	0/0/1	0/0/1	0/0/1	0/0/1	0/0/1	0/0/10
	Common areas	–	–	–	–	–	–	–	–	0/0/5	0/0/30
	Patient room 25^V^	6	Yes	**33.5**	0/1/2	0/0/1	0/1/0	0/1/0	0/1/0	**1**/0/0	0/1/9
	Common areas	–	–	–	–	–	–	–	–	0/0/6	0/0/30

SARS-CoV-2 results from environmental samples: Number of positive/inconclusive/negative. All positive samples from PCR are cultured from oropharyngeal swab, CDC-NIOSH bioaerosol sampler, SKC Bio-sampler, and surfaces wabs. Day of illness is counted since onset of symptoms. In case planned samples were not obtained the following reasons applied: retraction patient, no availability, or accessibility of area during sample collection, sample got lost, or discarded by staff.

*Living room was not available for sampling due to closure.

**Two patients on different wards.

***Cat supplies were sampled additionally.

● Duration of sampling 4 hr due to retraction patient/NO = 1 sample was not obtained/C = positive culture/AS = asymptomatic/OX = optiflow/V = vaccinated. In total 27 samples were not obtained because of retraction patient, no availability of area during sample collection, or discarded by staff.

### SARS-CoV-2 contamination in air

Of the 184 environmental samples, 82 air and 102 surface samples, collected in rooms of patients with positive OPS on the day of sampling, 50.5% were positive (93/184). From the two patients with negative OPS, only one of the samples tested positive (1/29), which appeared a surface swab (Table 1).

All four air sampling techniques detected SARS-CoV-2 RNA and showed high rates of positive samples in the rooms of patients with positive OPS (Table 3). The highest proportion of positive active air samples was found with the filtration-based sampler 80% (8/10) and CDC-NIOSH cyclone-based sampler [70% (7/10)]. The impingement-based sampler [50% (5/10)] showed a slightly lower proportion of positive samples, but the results were not statistically significant (Fisher-exact test *P*-value = 0.69). The cyclone-based samples sampled approximately 1.26 m^3^ of air, the filtration-based samples 1.26 m^3^, and the impingement-based 0.75 m^3^. Ten of the collected 12 settling dust samples from rooms were positive (83%). Filtration-based samples and cyclone-based samples collected side-by-side at the same distance from the patient were concordant in 8 out of 10 cases [moderate agreement (Cohen’s kappa coefficient kappa = 0.5, *P*-value = 0.197)] (Supplement Table S4, available at *Annals of Occupational Hygiene* online).

**Table 3.** SARS-CoV-2 results from three active and one passive air sampling technique used during the outbreak investigation from patients with positive oropharyngeal swab

	CIS- inhalable dust	SKC Bio-sampler - Impinger	CDC-NIOS Hcyclone- based bioaerosol *	EDC - settling dust
	*n* (%)	*n* (%)	*n* (%)	*n* (%)
Negative (‐ ‐)	9 (30)	4 (40)	2 (20)	2 (17)
Inconclusive (‐ +)	2 (7)	1 (10)	1 (10)	0 (0)
Positive (+ +)	19 (63)	5 (50)	7 (70)	10 (83)

*If one of the fractions of the cyclone-based sample detected SARS-CoV-2, the overall parameter is classified positive.

SARS-CoV-2 was detected at all distances from the patient (bedridden and mobile patients). No clear trend was seen in numbers of positive samples with distance from the patient in filtration-based air samples [>1.5 m 50% (6/12); ≤1.5 m 67% (10/15)] (Fisher-exact test, *P*-value = 0.4175) (Supplement Table S3, available at *Annals of Occupational Hygiene* online).

In all particle size-specific fractions [>4 μm 60% (6/10); 1–4 μm 50% (5/10); <1 μm 20% (2/10)] SARS-CoV-2 RNA was detected (Table 2). However, inconclusive and positive results were more frequently present in the largest particle size fraction, followed by the intermediate size fraction. These differences in distribution of size categories was borderline statistically significant (Fischer exact test *P*-value = 0.076).

**Table 2.** SARS-CoV-2 PCR results in size-specific fractions obtained by cyclone-based air sampling in rooms of patients with positive oropharyngeal swab

	CDC-NIOSH cyclone-based bioaerosol sampler		
	<1 μm	1–4 μm	>4 μm
	PTFE filter	Microcentrifuge tube 1.5 ml	Centrifuge tube 15 ml
	*n (%)*	*n (%)*	*n (%)*
Negative (‐ ‐)	8 (80)	4 (40)	2 (20)
Inconclusive (‐ +)	0 (0)	1 (10)	2 (20)
Positive (+ +)	2 (20)	5 (50)	6 (60)

### High- and low-touch surface swabs

The proportion of positive surface samples was much higher in rooms from patients with positive OPS compared to rooms with negative patients [43% (43/99) versus 0.5% (1/20)] [see Supplement Table S5 (available at *Annals of Occupational Hygiene* online)]. SARS-CoV-2 RNA was detected slightly more frequently in surface swabs from low-touch surfaces than from high-touch surfaces [low 50% (22/44); high 38% (21/55)] (Fisher’s exact test *P*-value = 0.18). Only 5 of the 197 surface samples collected in common areas were positive for SARS-CoV-2; four low and one high-touch sample (Supplement Table S6, available at *Annals of Occupational Hygiene* online).

### Virus culture

Among the 78 positive OPS, cyclone-based samples, impingement-based samples, surface swab samples, 44 had a RdRp Ct-value ≤35 and were further investigated by in vitro virus culture. This selection contained four impingement-based samples, three cyclone-based samples fraction size >4 μm, three cyclone-based samples fraction size 1–4 μm, 26 surface swabs, and eight OPS collected in nine patient rooms. The impingement-based samples and cyclone-based samples were collected in four patient rooms. Cytopathic effects were observed in three OPS and one active air sample and were confirmed by immunofluorescent staining. The active air sample from the CDC-NIOSH sampler (>4 µm size fraction) had the lowest Ct-value of all environmental samples (29.5) and was derived from the room of the patient with the lowest OPS Ct-value (19.82).

### Whole genome sequencing (WGS)

In total, nine samples with RdRp Ct-values ranging from 19.8 to 30.2 were selected for SARS-CoV-2 whole genome sequencing, of which six OPS, one cyclone-based sample, one filtration-based sample, and one surface swab. From five OPS samples, >90% of the reference was covered and uploaded in GISAID. All variants were B.1.221, a known variant, circulating in The Netherlands at the time of the study. Samples collected at the same location were closely genetically related. During the data collection from December 2020 until May 2021, B.1.1.7, also known as the Alpha variant, became the dominant SARS-CoV-2 circulating variant in The Netherlands (National Institute for Public Health and the Environment, 2022). The sequences have been registered in GISAID (www.gisaid.org; Accession ID EPI_ISL_2259112, EPI_ISL_2259136, EPI_ISL_2259188). See Supplementary Methods (available at *Annals of Occupational Hygiene* online) for more details see acknowledgement table.

### Longitudinal air monitoring

Only seven of the 259 settling dust samples collected repeatedly in three wards were positive (2.7%). All samples were collected in common areas in nursing homes where SARS-CoV-2 infections had been reported among residents (Supplement Table S7, available at *Annals of Occupational Hygiene* online). The low rate corroborates with the incidence of infections in patients and healthcare workers, which rapidly decreased during the study (Supplementary Table S7, available at *Annals of Occupational Hygiene* online). No viral RNA was detected in wards without registered SARS-CoV-2 infected patients and/or healthcare workers shortly before or during sampling.

## Discussion

In this study, comprising 679 environmental samples, SARS-CoV-2 was frequently detected in air and on surfaces in the immediate surroundings of COVID-19 patients, providing evidence of virus shedding to the environment through air by infected persons. SARS-CoV-2 was detected more frequently in the particle size fraction 1–4 μm (respirable fraction) and particulates >4 μm as compared to <1 μm. Airborne particulates might be infectious, as illustrated by the fact that we were able to replicate virus from an active air sample. Our results support the role of airborne transmission of SARS-CoV-2, which in turn is a risk factor for subsequent infection.

SARS-CoV-2 RNA was detected in all types of air samples and on high- and low-touch surfaces in the surrounding of patients with a positive OPS. No SARS-CoV-2 RNA was detected in air or the immediate surroundings of patients who tested negative. The number of positive environmental samples in this study was high compared to other studies (Lednicky *et al.*, 2020; Semelka *et al.*, 2021). Although the study size is small to modest, environmental sampling was performed extensively, using a range of sampling techniques, around patients in the early phase of infection, assuming active shedding of SARS-CoV-2. Previously, van Beek *et al.* (2021), established a shedding curve using data from 223 persons testing SARS-CoV-2 in a drive-through test station, showing that viral loads were highest within eight days post-onset of symptoms. Moreover, Van Kampen *et al.* (2021), reported that infectious virus shedding also occurred mainly within the first eight days post-onset, based on data from 129 hospitalized patients with repeated measurements. Therefore, our study’s timing of environmental measurements has likely contributed to the high detection rate in environmental samples. This is in agreement with a study from Chia *et al.* (2020) only detecting SARS-CoV-2 in air or the immediate surroundings of two patients infected less than eight days compared to no detection of SARS-CoV-2 in the air of another patient nine days postinfection. Several other studies were not able to detect SARS-CoV-2 in air in the surrounding of patients more than eight days after post-onset of symptoms (Chia *et al.*, 2020; Dumont-Leblond *et al.*, 2021; Semelka *et al.*, 2021). Moreover, in other studies in human and animal settings, SARS-CoV-2 was only detected in environmental samples if the human and animal source organisms were actively shedding SARS-CoV-2 during sampling (de Rooij *et al.*, 2021; Jonker *et al.*, 2022). These observations emphasize that timing of sampling in the direct environment of patients and other populations is of importance for detecting SARS-CoV-2 and that surroundings from SARS-CoV-2 patients in early stage are contaminated with SARS-CoV-2.

Of the SARS-CoV-2 containing aerosols, 54% was in the size range <4 µm and 46% in the size range of ≥4 µm. When including samples with inconclusive qPCR test results, these figures hardly changed (50–50%). Although the use of the NIOSH sampler was modified by adding VTM to the vials prior to sampling, which may theoretically have altered size-selective sampling characteristics, our results are in line with other studies that performed size-selective sampling of SARS-CoV-2 virus. For instance, Adenaiye *et al.* (2021), analysed SARS-CoV-2 virus in exhaled breath collected from 49 COVID-19 cases (mean days postonset 3.8 ± 2.1) in an experimental setting and found SARS-CoV-2 RNA in 36% of fine (≤5 µm), and 26% of coarse (>5 µm) aerosols. Moreover, other studies using the same CDC-NIOSH bio-sampler methodology as this study, exclusively detected SARS-CoV-2 in the larger ≥4 µm and intermediate size fraction 1–4 µm in environmental samples collected in rooms of COVID-19 patients in hospitals (Chia *et al.*, 2020; Conway-Morris *et al.*, 2021). Similar observations in size distribution have been reported previously for human influenza virus (Blachere *et al.*, 2009; Lindsley *et al.*, 2010). These results for different viruses from infected patients indicate that a substantial part of particulates is found in the respirable fraction (Blachere *et al.*, 2009). Viral RNA loads and infectious viral RNA loads can differ between patients and are likely influenced by infection status and disease progression. Moreover, the strain-specific viral load and the location of infection in airways influence the particle size distribution and transmission mode to the environment. A different variant, such as Omikron, which is more contagious and is primarily present in the upper respiratory tract, might therefore distribute differently in the environment (Vihta *et al.*, 2022).

Out of ten active air samples eligible for culture, we were able to replicate virus from one sample. Only a few studies successfully showed signs of SARS-CoV-2 replication in air samples (Lednicky *et al.*, 2020; Santarpia *et al.*, 2020; Adenaiye *et al.*, 2021). However, underestimation of infectiousness is a likely consequence of virus inactivation during sample collection (Lindsley *et al.*, 2010). Current culture techniques may not be optimal for low viral concentrations as in air samples (Zhang *et al.*, 2020). Overall, results suggest that virus particulates can cause infection in individuals who inhale these particulates when the infectious dose is sufficiently high.

Literature on the infectious dose of SARS-CoV-2 is scarce. Dabisch *et al.* (2021) reported an infectious dose of 52 TCID_50_ for a seroconversion response and 256 TCID_50_ for a fever response based on an inhalation exposure of 10 min in nonhuman primates Macaques. Others have estimated an infectious dose for infection ranging between single and 1000 virions based on a model combining information on viral mutations obtained through deep sequencing and epidemiology in known infector-infectee pairs (Popa *et al.*, 2020; Martin and Koelle, 2021; Nicholson *et al.*, 2021). Based on the estimated relationship between E-gene RT-PCR Cq values and cell-cultured SARS-CoV-2 virus loads by Schuijt *et al.* (2021), the air sample which showed replication in our study contained approximately 170 000 viral copies per cubic meter of air. Despite uncertainties associated with this simple calculation (for instance, assuming similarity in RT-qPCR responses between cell-cultured virus and air samples), the estimated dose may indeed be capable of causing infection. Quantification of the other environmental samples was not attempted, since uncertainty would even be greater due to high Ct-values. Moreover, our measurements took place during relatively long periods. Environmental levels likely varied considerably over the sampling period. Variation in viral load could not be established over this time span. However, it is unlikely that viral shedding is constant over time. Coughing, for instance, results in higher viral RNA loads over a short time span.

There is an ongoing debate on the airborne transmission route of SARS-CoV-2 and the effect of ventilation on airborne transmission. Greenhalg *et al.* (2021) previously pointed out multiple reasons for airborne transmission as the main route of SARS-CoV-2, to which our study provides additional strength. First, our study detected SARS-CoV-2 in abundance in air and on surfaces, including numerous low-touch surfaces such as on top of the wardrobe, which implicates viral dissemination through the air by aerosols. Second, SARS-CoV-2 was primarily found in particle size fractions of 1–4 μm and larger than 4 μm, which are known to stay airborne for extended periods of time and thus disseminate potentially over larger distances. Third, we successfully cultured SARS-CoV-2 from an active air sample from particle size >4 μm and aerosols have been reported to stay infectious in the air for up to 3 hr (van Doremalen *et al.*, 2020).

Based on our study, smaller particles (<1 μm), which can travel further, do not seem to be the key vehicle of SARS-CoV-2 transmission. Although virus contamination was omnipresent in air in infected patient rooms, the vast majority of settling dust and surface swab samples from common areas were negative, suggesting SARS-CoV-2 transmission is more a local phenomenon than widespread. To mitigate (occupational) transmission risks, it is important to investigate the effect of ventilation and air filtration on airborne transmission reduction. Till date, only Conway-Morris *et al.* (2021) investigated and successfully demonstrated removal of SARS-CoV-2 from air by placing active filtration and sterilization devices in wards. Further research on the effect of ventilation and filtration devices is required to draw strong conclusions about the role of ventilation conditions in reducing airborne transmission. Despite the aforementioned limitations of this study, such as sample size and semi-quantitative results, SARS-CoV-2 is detected regularly confirming the potential airborne transmission route of SARS-CoV-2 for subsequent infection. Replication of this study in a larger sample size is required to investigate dispersal abilities, infectiousness, and particle sizes of aerosol containing SARS-CoV-2.

In conclusion, in this study potential airborne transmission routes from SARS-CoV-2 infected patients to their immediate surroundings were investigated. SARS-CoV-2 was numerously detected in air and on surfaces in case of actively shedding patients. Furthermore, the environmental contamination of SARS-CoV-2 and infectious aerosols confirm the potential for airborne transmission routes via air up to several meters and therefore the possible risk of infection of SARS-CoV-2. These insights can contribute to the discussion on airborne transmission and facilitate effective design of prevention strategies such as use of facemasks and optimising ventilation conditions.

## Supplementary Material

wxac056_suppl_Supplementary_Material

## Data Availability

The data underlying this article will be shared on reasonable request to the corresponding author.

## Creative commons

This is an open access article distributed in accordance with the Creative Commons Attribution Non Commercial (CC BY-NC 4.0) license.
