# Supplementary material for: Detection of SARS-CoV-2 in Air and on Surfaces in Rooms of Infected Nursing Home Residents
Source: Ann Work Expo Health. 2022 Sep 7;67(1):129–40. doi: 10.1093/annweh/wxac056 (PMC9834894; doi:10.1093/annweh/wxac056)
Supplement: wxac056_suppl_Supplementary_Material [file wxac056_suppl_supplementary_material.docx]

**Detection of SARS-CoV-2 in air and on surfaces in rooms of infected nursing home residents**

*K.J. Linde^1^, I.M. Wouters^1^,* *J.A.J.W. Kluytmans^5^, M.F.Q. Kluytmans-van den Bergh^5,6^, S.D. Pas^3^, C.H. GeurtsvanKessel^2^, M.P.G. Koopmans^2^, M. Meier^4^, P. Meijer^1^, C.R. Raben^1^, J. Spithoven^1^, M.H.G. Tersteeg-Zijderveld^1^, D.J.J. Heederik^1^, W. Dohmen^1^ and COCON consortium*

*^1^Institute for Risk Assessment Sciences, Utrecht University, Utrecht, the Netherlands*

*^2^Department of ViroScience, Erasmus MC, Rotterdam, the Netherlands*

*^3^Microvida location Amphia/Bravis, Breda/Roosendaal, the Netherlands*

*^4^Mijzo, Waalwijk, the Netherlands*

*^5^Julius Center for Health Sciences and Primary Care, University Medical Center Utrecht, Utrecht University, Utrecht, the Netherlands*

*^6^Department of Infection Control, Amphia Hospital, Breda, the Netherlands*

Corresponding author: K J Linde

e-mail: K.j.linde@uu.nl

Institute for Risk Assessment Sciences

Division Environmental Epidemiology

PO Box 80178

3508TD Utrecht

The Netherlands

**Supplement: method and materials**

**Outbreak investigation**

*Filtration-based sampler*

The filtration-based technique captures inhalable dust - airborne particles and droplets of an aerodynamic size that enter the respiratory tract through mouth and nose. Air is drawn through an inhalable dust sampling head (Conical Inhalable Sampler, JS Holdings, Stevenage, UK) equipped with 37mm diameter 2.0 μm pore-size Teflon filter (Pall incorporated, Ann Arbor, USA) connected with tubing to a Gilian GilAir 5 pump (Sensidyne, St. Petersburg, USA) calibrated at a flow of 3.5 L/min. The sampling head were attached to a pole at 1.5m height as this is average breathing height. The sampling train was set up before the beginning of the measurement and lasted for 6 hours. In each patient room, three inhalable dust samplers were placed, one near the head of the patient, one near the feet of the patient and one near the location often used by healthcare worker (Figure S1).

*Cyclone-based sampler*

The cyclone-based technique allowed for size-selective sampling of dusts and aerosols from the environment. The CDC-NIOSH cyclone-based bioaerosol sampler (NIOSH BC 251, kindly provided to us by William G. Lindsley, NIOSH Morgantown, USA) consists of a sampling body which is equipped with a 15ml conical tube (Greiner Bio-One, Alphen aan de Rijn, Netherlands) to capture aerosols larger than 4 μm in size, a 1.5ml conical tube (Sarstedt BV, Etten-Leur, Netherlands) to capture aerosols with a size ranging between 1-4 μm, and a 37mm diameter filter holder (SKC Incorporated, Eighty Four, USA) equipped with 37mm diameter 2.0 μm pore-size Teflon filter (Pall incorporated, Ann Arbor, USA) to collect particles of 1 μm and smaller. The filter holder was connected with tubing to a Gilian GilAir 5 pump (Sensidyne, St. Petersburg, USA) calibrated at a flow of 3.5 L/min. The 15ml and 1.5ml tube were pre-filled with 2.5ml and 1ml virus transport medium 1 (VTM-1; Erasmus Medical Center (EMC), Rotterdam, The Netherlands), respectively, before start of the measurement (table S1 composition of media). The sampling train was set up before the beginning of the measurement and lasted for 6 hours. In each patient room, one cyclone sampler was placed in conjunction with one of the filtration-based samplers close to the feet of the patient. After sampling filter holders were detached, packed in a Minigrip^TM^ bag. The 15ml and 1.5ml tubes were detached from the sampling body, 1 ml Opti-MEM^TM^ (Gibco, UK) and 1 ml of VTM-1/Opti-MEM^TM^ mixture, respectively, was added to the tubes immediately after sampling.

*Impingement-based sampler*

Air sampling through impingement was conducted by means of a 5ml BioSampler (SKC Inc, Eighty Four, USA) positioned at 1.5m height attached to a pole. An airflow of 12.5 L/min through the BioSampler was established by connecting the outlet of the sampler to an inhouse designed pump unit. The impinger was filled with 4ml of VTM-1 used for air sampling (see above) prior to the beginning of the measurement. The measurement lasted 1 hour close to the patient’s head, during which evaporation losses of VTM was replaced by adding every 15 minutes 2 ml of VTM-1. After sampling, remaining VTM fluid was transferred to a 15ml tube (Greiner BioOne, Etten-Leur, Netherlands) and 2 ml of Opti-MEM^TM^ was added.

*Settling dust sampler*

Settling dust, passive air, samples were collected by using Electrostatic Dust Collectors (EDCs) (Noss et al., 2008), which were placed in each included patient room and corresponding hallway, common living room and nurse office of the ward. EDCs were placed in holders pinned to the ceiling in the middle of the space. After 2-4 weeks of sampling EDCs were picked up and packed in a Minigrip^TM^ bag for transportation.

*Collection of surface samples*

In each of the above areas: each included patient room, hallway, living room and nursing office, ten swab samples from surfaces were collected. Sampling locations included high-touch surfaces like door handle and tabletop and low-touch surfaces like top surface of cabinets. To standardize swabbing of surfaces, disposable plastic grids of 10 cm^2^ were used; when it was not possible to use the grid this was noted. Dry swabs (Medical Wire Dry Swabs, MW370, Corsham, UK) were used, which were placed in 2ml viral transport medium 2 (VTM-2; Erasmus Medical Center (EMC), Rotterdam, The Netherlands) in 5ml tubes directly after swabbing (for composition of media see table S2).

*Patient characteristics*

Oropharyngeal swabs (OPS) was collected from patients during sample collection. Dry swabs (Medical Wire Dry Swabs, 111598, Milano, Italy) were used, which were placed in 2ml VTM-2 in 5ml tubes directly after swabbing.

**Laboratory analysis**

After collection, all samples, except for EDCs, were stored at 4 °C in electric transport cooling box and transported to the lab. At the lab, all samples, except for EDCs, were placed in 4°C storage until further processing the next day. All handlings were performed under BSL2+ conditions. In short, filters were removed from the filter holders and transferred to 5ml screw-top tubes (Eppendorf, Nijmegen, Netherlands) and 2ml of VTM-1 used for air sampling was added, next tubes were vortexed for 5 minutes using a vortex adaptor. Tubes containing VTM and Opti-MEM^TM^ mixture from NIOSH and impingement-based samplers and OPS were vortexed for 15 seconds, and tubes containing the surface swab samples were vortexed for 1 min prior to further handling. After vortexing 600 μl was transferred and added to a tube containing 600 μl of MagNA Pure 96 External Lysis Buffer (Roche Diagnostics, Almere the Netherlands), followed by 15 seconds of vortexing. Aliquots with remaining VTM from NIOSH, impingement, surface swab and OPS were stored frozen at -80 ℃ for culturing. Aliquots with VTM/lysis buffer samples were stored frozen at -80 ℃ for PCR.

*Settling dust sampler*

EDCs were transferred to tube containing 10 ml VTM-2. EDCs were tamped down with disposable pipette, followed by repeating twice 15 seconds of vortexing and soaked for 15 minutes and ended with 15 minutes of vortexing. EDCs were tamped down with disposable pipette and 600 ul sample was transferred and added to a tube containing 600 ul MagNA Pure 96 External Lysis Buffer (Roche Diagnostics, Almere the Netherlands) and followed by 15 seconds of vortexing. VTM/lysis buffer samples were stored frozen at -80 ℃.

*Real time quantitative RT-PCR*

Samples were transported on dry ice to Microvida Laboratory for Medical Microbiology, Bravis Hospital, Roosendaal, The Netherlands. Presence of SARS-CoV-2 RNA was tested using a SARS-CoV-2 RNA RT-qPCR, targeting the E gene and CoV-2 RdRP-gene of SARS-CoV-2. The cobas® SARS-CoV-2 Test (Roche Diagnostics, Basel, Switzerland) was performed on the samples with lysis buffer using the cobas® 6800 Systems (Roche Diagnostics). Samples were positive if the threshold was below Ct-value 40.

**SARS-CoV-2 Whole genome sequencing (WGS)**

Samples with RT-PCR RdRp Ct-values <31 whole genome sequencing of the primary clinical specimen was performed by Microvida to determine the SARS-CoV-2 variant. In short, total nucleic acids were extracted using the QIAsymphony DSP virus pathogen midi kit and pathogen complex 400 protocol of the QIAsymphony Sample Processing system (Qiagen, Germany), with an input volume of 400 μL and output volume of 110 μL. cDNA was synthesized using LunaScript® RT SuperMix Kit (New Engeland Biolabs, USA) and library preparation was performed using EasySeqTM RC-PCR SARS-CoV-2 Whole Genome Sequencing kit (Nimagen, The Netherlands) according to manufacturer’s instructions. Subsequent next generation sequencing (NGS) of 2x150cycles paired end reads was performed on a Miseq (Illumina, The Netherlands) using MiSeq Reagent Micro Kit v2 according to manufacturer’s instructions. Data analyses was performed with an in-house workflow using CLCbio Genomic Workbench v21 (Qiagen, Germany), including (a.o.) read-trimming, NC_045512.2 (NCBI genbank) reference-based assembly, local re-alignment and variant detection algorithms. The consensus genome was extracted and positions with a coverage less than 10 reads were replaced with N. The sequences were manually curated. Genomes with >70% genome coverage were included for lineage assignment using Pangolin (<https://pangolin.cog-uk.io/>)(O’Toole et al. 2021) and Nextclade Web ([https://clades.nextstrain.org/) (Hadfield et al., 2018))](https://clades.nextstrain.org/)%20(Hadfield%20et%20al.,%202018))). Genomes with >90% genome coverage were uploaded to GISAID (<https://www.gisaid.org/>)(Elbe and Buckland-Merrett 2017), with accession IDs EPI_ISL_3047866, EPI_ISL_3047867, EPI_ISL_2259188, EPI_ISL_2259136 and EPI_ISL_2259122 and phylogenetic analysis performed using MAFFT alignment software (Galaxy Version 7.221.3, FFT-NS method) (Katoh and Standley 2013) and phylogenomic software IQ-TREE 2.1.3 (Galaxy Version 1.5.5.3) (Kalyaanamoorthy et al. 2017; Minh et al. 2020; Minh, Nguyen, and von Haeseler 2013), using ModelFinder (with best predicted method GTR+F+I), ultrafast bootstrapping (1000 replicates) and maximum likelihood tree reconstruction. Phylogenetic tree was visualized in CLCbio Genomic Workbench v20.

*Virus culture*

Positive tested samples with RT-qPCR from the OPS, cyclone-based, impingement-based and surface swabs were transported on dry ice, using the duplex aliquot without lysis buffer for culturing at Erasmus University Medical Centre laboratory, Rotterdam, the Netherlands. Culturing was performed on Vero cells, clone 118 at 37°C, and 5% CO_2_ and was completed after 7 days. If virus-induced cytopathic effect was observed, immunofluorescent detection of nucleocapsid proteins was performed to confirm the presence of SARS-CoV-2 (van Kampen et al. 2021).

*Modification in sample collection*

After the first outbreak investigation outbreak A, an optimised protocol was implemented. For the first outbreak investigation VTM-2 was used as pre-fill in the tubes for the cyclone-based, impingement-based and re-fill for impingement sampling in instead of VTM-2. After sampling the impingement-based sampler VTM fluid was transferred to a 15 ml tube (Greiner BioOne, Etten-Leur, Netherlands) containing 1,5 ml Fetal Bovine Serum (FSB) (40 v/v%, Greiner Bio-One, Etten-Leur, Netherlands). Instead of adding Opti-MEM^TM^ mixture or VTM/Opti-MEM^TM^ mixture after sampling to the cyclone-based samples, the day after during processing only VTM-2 was added up to the original amount prior to sample collection. Sampling and processing of surface swabs, OPS and EDCs stayed identical.

**References**

Elbe S, and Buckland-Merrett G. 2017. “Data, Disease and Diplomacy: GISAID’s Innovative Contribution to Global Health.” *Global Challenges* 1(1):33–46. doi: 10.1002/gch2.1018.

Kalyaanamoorthy S, Minh BQ, Wong TKF, von Haeseler A, and Jermiin LS. 2017. “ModelFinder: Fast Model Selection for Accurate Phylogenetic Estimates.” *Nature Methods* 14(6):587–89. doi: 10.1038/nmeth.4285.

Katoh K, and Standley DM. 2013. “MAFFT Multiple Sequence Alignment Software Version 7: Improvements in Performance and Usability.” *Molecular Biology and Evolution* 30(4):772–80. doi: 10.1093/molbev/mst010.

Minh BQ, Nguyen MAT, and von Haeseler A. 2013. “Ultrafast Approximation for Phylogenetic Bootstrap.” *Molecular Biology and Evolution* 30(5):1188–95. doi: 10.1093/molbev/mst024.

Minh BQ, Heiko A. Schmidt, Chernomor O, Schrempf D, Woodhams MD, von Haeseler A, Lanfear R, and Teeling E. 2020. “IQ-TREE 2: New Models and Efficient Methods for Phylogenetic Inference in the Genomic Era.” *Molecular Biology and Evolution* 37(5):1530–34. doi: 10.1093/molbev/msaa015.

Noss I, Wouters IM, Visser M, Heederik DJJ, Thorne PS, Brunekreef B, and Doekes G. 2008. “Evaluation of a Low-Cost Electrostatic Dust Fall Collector for Indoor Air Endotoxin Exposure Assessment.” *Applied and Environmental Microbiology* 74(18):5621–27. doi: 10.1128/AEM.00619-08.

O’Toole Á, Scher E, Underwood A, Jackson B, Hill V, McCrone JT, Colquhoun R, RuisC, Abu-Dahab K, Taylor B, Yeats C, du Plessis L, Maloney D, Medd N, Attwood SW, Aanensen DM, Holmes EC, Pybus OG, and Rambaut A. 2021. “Assignment of Epidemiological Lineages in an Emerging Pandemic Using the Pangolin Tool.” *Virus Evolutio*n 2021 Jul 30;7(2):veab064. doi: 10.1093/ve/veab064.

*
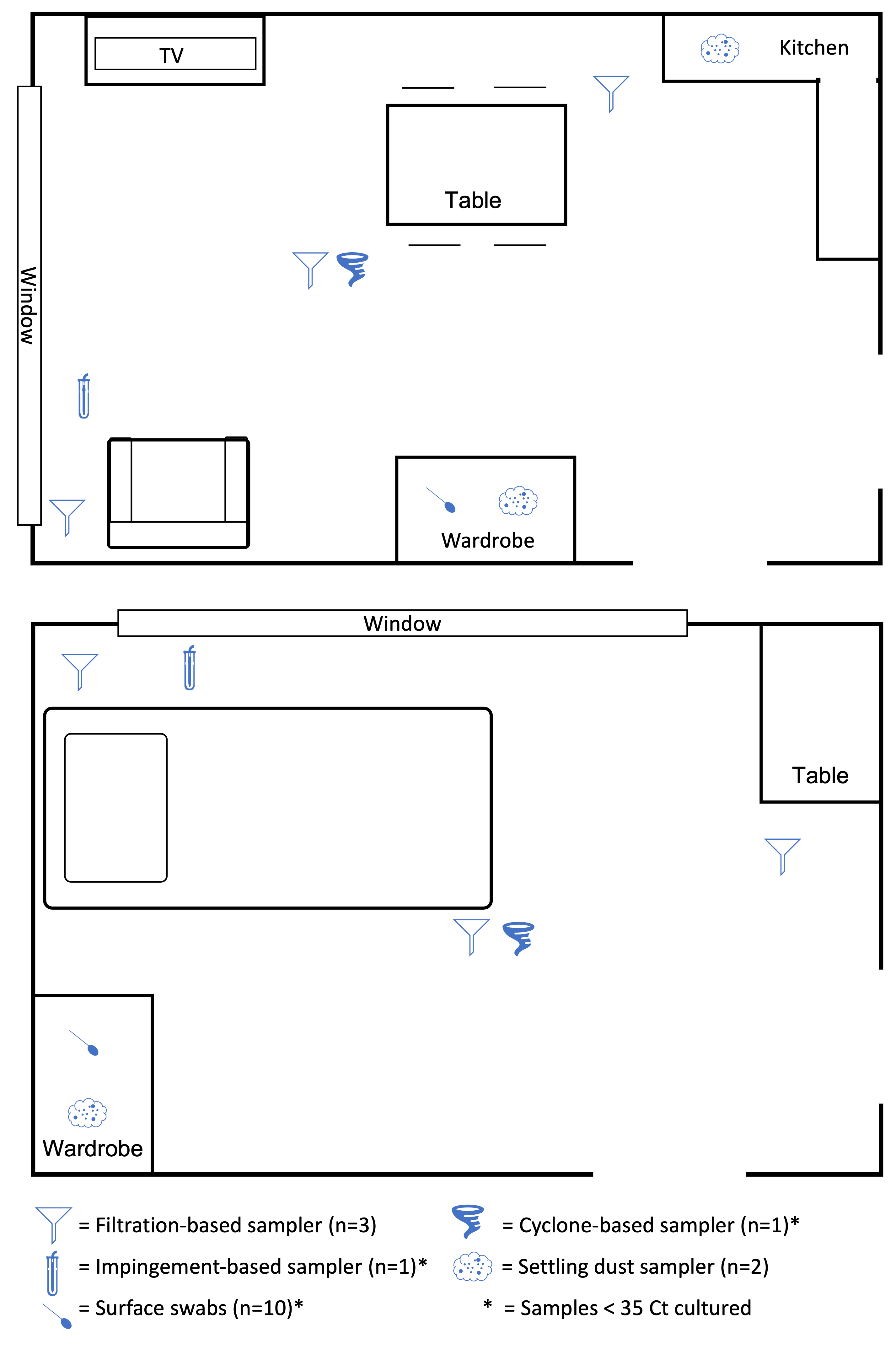
*

**Figure S1: Illustration of sample collection in patient room.** The first figure shows data collection in the living room of a mobile patient sitting in a chair. The second figure illustrates data collection in the sleeping room of a bedridden patient.

**Table S1: VTM-1 composition**

| **Name** | **Volume (ml)** | **Brand** | **Cat. Nr.** |
| --- | --- | --- | --- |
| HMEM (with 25 mM Hepes) | 4000 mL | Lonza/Westburg BV | LO 12-137F |
| HEPES, 1M | 700ml | Lonza/Westburg BV | BE17-737E |
| Glycerol 99% | 480 ml | Sigma Aldrich | G6279-1L |
| Lactalbumin enzymatic hydrolysate** | 20 gr | Sigma Aldrich | 61300-500G |
| Polymyxin B Sulphate (1,67mg/ml) | 48 ml | Sigma Aldrich | P0972-10MU |
| Nystatin (1,69mg/ml) | 24 ml | Sigma Aldrich | N6261-5MU |
| Pen/Strep (10.000U/10.000uG) | 96 ml | Westburg | LO DE17-602E |
| Gentamicin | 24 ml | Life Technologies | 15750037 |

**Table S2: VTM-2 composition**

| **Name** | **Volume (ml)** | **Brand** | **Cat. Nr.** |
| --- | --- | --- | --- |
| Dulbecco’s Modified Eagle’s Medium (DMEM)  (without NaHCO3 Hepes, L-Glutamin) | 107 | Lonza | BESP070 |
| Penicillin/Streptomycin   (10000 U/ml; 10000 µg/ml) | 25 | Lonza | 17-602E |
| Amphotericin B (0,25 mg/ml) | 10 | Pharmacy, EMC | n.a. |
| NaHCO3 | 3 | Lonza | 17-613E |
| Hepes (1M) | 5 | Lonza | 17-737E |
| Fetal Bovine Serum (FBS) | 100 | Greiner Bio-one | 758093 |

**Supplement: Results**

**Table S3: SARS-CoV-2 PCR results in inhalable dust samples collected in rooms of patients with positive OS stratified by distance to the patient**

|  | **CIS – inhalable dust** | |
| --- | --- | --- |
|  | **<1,5 N=15** | **>1,5 meter N=12** |
| Negative (- -) | 5 (33) | 4 (33) |
| Inconclusive (- +) | 0 (0) | 2 (17) |
| Positive (+ +) | 10 (67) | 6 (50) |

During data collection of one patient no distances were obtained (n=3).

**Table S4: SARS-CoV-2 PCR results in inhalable dust samples cyclone-based samples from patients with positive OS**

|  | **CIS -  inhalable dust** | **CDC-NIOSH bioaerosol sampler -  cyclone-based** | **Distance (cm)** |
| --- | --- | --- | --- |
| Patient room 10 | **+ +** | **+ +** | NA |
| Patient room 12 | **+ +** | **+ +** | 160 |
| Patient room 13 | **+ +** | **+ +** | 100 |
| Patient room 15 | - - | - - | 210 |
| Patient room 16 | **+ +** | **+ +** | 85 |
| Patient room 17 | **+ +** | **+ +** | 74 |
| Patient room 18 | **+ +** | **+ +** | 140 |
| Patient room 20 | **+ +** | **+ +** | 135 |
| Patient room 22 | **+ +** | - - | 100 |
| Patient room 25 ^V^ | - - | - + | 130 |

V = fully vaccinated patient

**Table S5: SARS-CoV-2 PCR results surface swab samples from patients with positive OS**

|  | **High-touch** | **Low-touch** |
| --- | --- | --- |
| Negative (- -) | 33 (60) | 19 (43) |
| Inconclusive (- +) | 1 (2) | 3 (7) |
| Positive (+ +) | 21 (38) | 22 (50) |

**Table S6: SARS-CoV-2 PCR results surface swab samples from common areas**

|  | **High-touch** | **Low-touch** |
| --- | --- | --- |
| Negative (- -) | 94 (98,9) | 98 (96) |
| Inconclusive (- +) | 0 (0) | 0 (0) |
| Positive (+ +) | 1 (1,1) | 4 (4) |

**Table S7: SARS-CoV-2 PCR in environmental samples collected at nursing homes wards and reported SARS-CoV-2 infections from corresponding nursing homes**

|  | **Period 1** | | **Period 2** | | **Period 3** | | **Period 4** | |
| --- | --- | --- | --- | --- | --- | --- | --- | --- |
| **Wards** | **EDC –**  **Settling dust** | **Infections** | **EDC –**  **Settling dust** | **Infections** | **EDC –**  **Settling dust** | **Infections** | **EDC –**  **Settling dust** | **Infections** |
| m | **2**/1/3 | **>= 3 ^P^** | 0/1/5 | 0 | 0/0/6 | 0 | 0/0/6 | 0 |
| n | 0/0/6 | NO | 0/0/6 | 0 | 0/0/6 | 0 | 0/0/6 | 0 |
| o | 0/0/4 | NO | 0/0/4 | 0 | 0/0/4 | 0 | 0/0/4 | 0 |
| p | 0/0/6 | NO | 0/0/6 | 0 | 0/0/6 | 0 | 0/0/6 | 0 |
| q* | 0/1/1 ^NO^ | NO | 0/0/2 | 0 | 0/0/2 | 0 | 0/0/2 | 0 |
| r | **3**/0/2 | **>= 6 ^P^** | 0/1/4 | 0 | 0/0/5 | 0 | 0/0/5 | 0 |
| s | 0/0/5 | **>= 1 ^P^** | 0/0/5 | **2 ^P^** | 0/0/5 | **1 ^P^** | 0/0/5 | 0 |
| t | 0/0/6 | NO | 0/0/6 | 0 | 0/0/6 | 0 | 0/0/6 | 0 |
| u | **2**/2/2 | NO**** | 0/0/6 | 0 | 0/0/6 | 0 | 0/0/6 | 0 |
| v | 0/0/6 | 0 | 0/0/6 | 0 | 0/0/6 | 0 | 0/0/6 | 0 |
| w | 0/0/5 | 0 | 0/1/4 | 0 | 0/0/5 | 0 | 0/0/5 | 0 |
| x | 0/0/6 | **1 ^H^** | 0/0/6 | **1 ^H^** | 0/0/6 | 0 | 0/0/6 | 0 |
| y** | NO | NO | NO | NO | NO | NO | NO | NO |
| z*** | 0/0/6 | **1 ^P^** | NO | NO | NO | NO | NO | NO |

SARS-CoV-2 results from environmental samples: Number of positive/inconclusive/negative / * = two extra settling dust samples in general common areas included: restaurant and hairdresser / ** = no settling dust samples in common area collected / *** = only during outbreak measurement settling dust samples collected in common area /**** = shortly prior to the data collection a SARS-CoV-2 outbreak occurred at the ward/ P = confirmed PCR SARS-CoV-2 infection of patient / H = confirmed PCR SARS-CoV-2 infection of health caretaker / >= = not all infections were reported /NO = sample/information was not obtained.


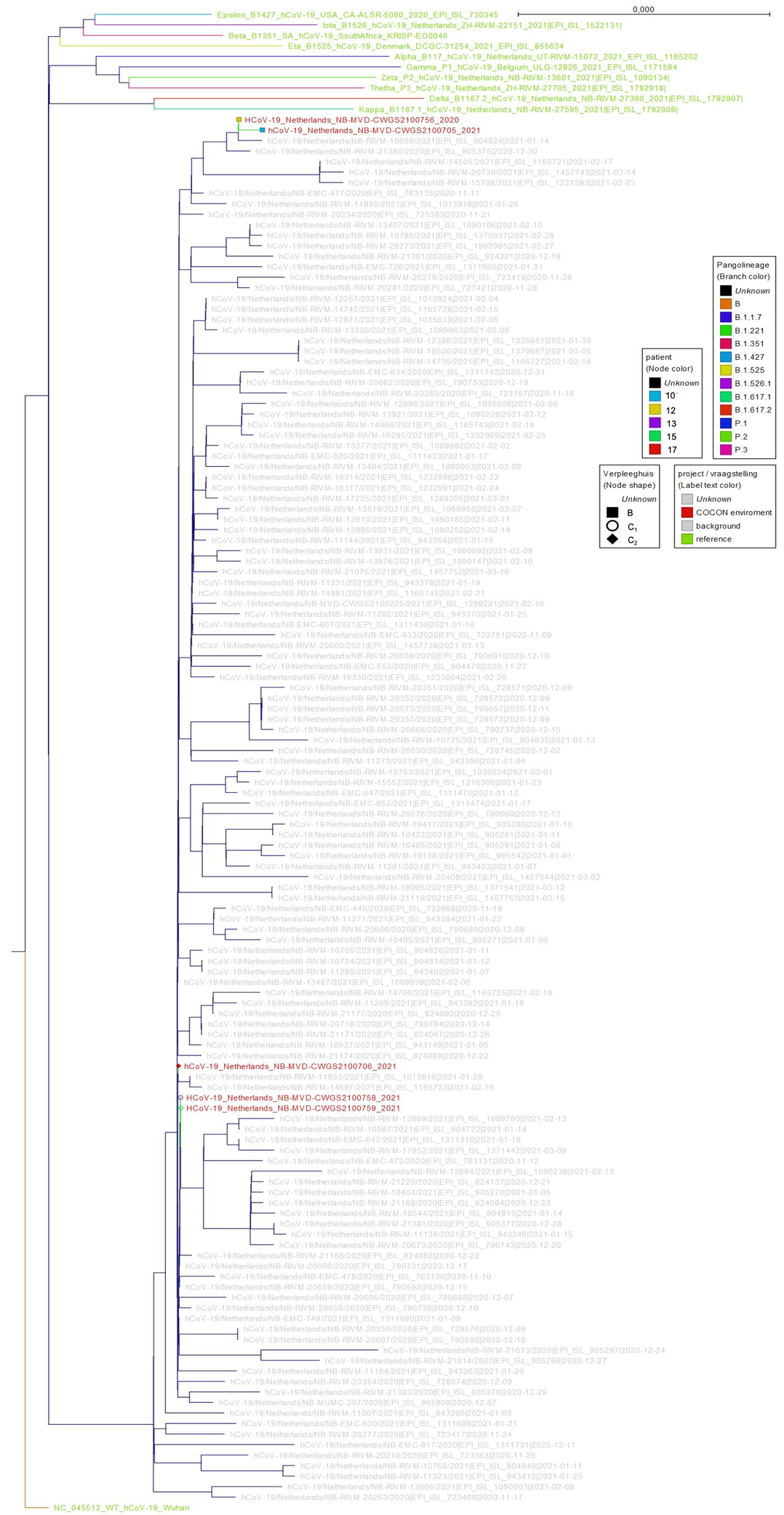


**Figure S2:** A phylogenetic analysis of samples with minimum >90% reference coverage.

**Table S8: Acknowledgement table Whole Genome Sequencing**

We gratefully acknowledge the following Authors from the Originating laboratories responsible for obtaining the specimens, as well as the Submitting laboratories where the genome data were generated and shared via GISAID, on which this research is based. All Submitters of data may be contacted directly via [www.gisaid.org](http://www.gisaid.org) Authors are sorted alphabetically.

| **GISAID accession number** | **Originating lab** | **Submitting lab** | **Authors** |
| --- | --- | --- | --- |
| EPI_ISL_1165202 | Dutch COVID-19 response team | National Institute for Public Health and the Environment (RIVM) | Adam Meijer, Harry Vennema, Dirk Eggink, Jeroen Cremer, Sharon van den Brink, Bas van der Veer, AnneMarie van den Brandt, Florian Zwagemaker, Dennis Schmitz, Chantal Reusken, on behalf of the national COVID-19 response team |
| EPI_ISL_736953 | NHLS-IALCH | KRISP, KZN Research Innovation and Sequencing Platform | Giandhari J, Pillay S, Lessells R, ChimukangaraB, Mdlalose K, York D, Khan S, Tegally H, Wilkinson E, de Oliveira T |
| EPI_ISL_1792907 | Dutch COVID-19 response team | National Institute for Public Health and the Environment (RIVM) | Adam Meijer, Harry Vennema, Dirk Eggink, Jeroen Cremer, Sharon van den Brink, Bas van der Veer, AnneMarie van den Brandt, Florian Zwagemaker, Dennis Schmitz, Chantal Reusken, on behalf of the national COVID-19 response team |
| EPI_ISL_730345 | San Diego County Public Health Laboratory | Andersen lab at Scripps Research | SEARCH Alliance San Diego with Tracy Basler, Jovan Shephard, Brett Austin |
| EPI_ISL_855634 | Department of Virus and Microbiological Special Diagnostics, Statens Serum Institut, Copenhagen, Denmark | Aalborg University | Danish Covid-19 Genome Consortium |
| EPI_ISL_1171584 | I.F.A.C. Hopital Princesse Paola | GIGA Medical Genomics | Keith Durkin, Maria Artesi, Sébastien Bontems, Raphaël Boreux, Bouchra Boujemla, Nathalie Renotte, Cécile Meex, Pierrette Melin, Marie-Pierre Hayette, Vincent Bours |
| EPI_ISL_722751 | Dutch COVID-19 response team | Erasmus Medical Centre | Bas Oude Munnink, Reina Sikkema, David Nieuwenhuijse, Irina Chestakova, Anne van der Linden, Marjan Boter, Emmanuelle Munger, Corine GeurtsvanKessel, Annemiek van der Eijk, Richard Molenkamp, Marion Koopmans, on behalf of the Dutch national COVID-19 response team. |
| EPI_ISL_722668 | Dutch COVID-19 response team | Erasmus Medical Centre | Bas Oude Munnink, Reina Sikkema, David Nieuwenhuijse, Irina Chestakova, Anne van der Linden, Marjan Boter, Emmanuelle Munger, Corine GeurtsvanKessel, Annemiek van der Eijk, Richard Molenkamp, Marion Koopmans, on behalf of the Dutch national COVID-19 response team. |
| EPI_ISL_763131 | Dutch COVID-19 response team | Erasmus Medical Centre | Bas Oude Munnink, Reina Sikkema, David Nieuwenhuijse, Irina Chestakova, Anne van der Linden, Marjan Boter, Emmanuelle Munger, Corine GeurtsvanKessel, Annemiek van der Eijk, Richard Molenkamp, Marion Koopmans, on behalf of the Dutch national COVID-19 response team. |
| EPI_ISL_763135 | Dutch COVID-19 response team | Erasmus Medical Centre | Bas Oude Munnink, Reina Sikkema, David Nieuwenhuijse, Irina Chestakova, Anne van der Linden, Marjan Boter, Emmanuelle Munger, Corine GeurtsvanKessel, Annemiek van der Eijk, Richard Molenkamp, Marion Koopmans, on behalf of the Dutch national COVID-19 response team. |
| EPI_ISL_763136 | Dutch COVID-19 response team | Erasmus Medical Centre | Bas Oude Munnink, Reina Sikkema, David Nieuwenhuijse, Irina Chestakova, Anne van der Linden, Marjan Boter, Emmanuelle Munger, Corine GeurtsvanKessel, Annemiek van der Eijk, Richard Molenkamp, Marion Koopmans, on behalf of the Dutch national COVID-19 response team. |
| EPI_ISL_904470 | Dutch COVID-19 response team | Erasmus Medical Centre | Bas Oude Munnink, Reina Sikkema, David Nieuwenhuijse, Irina Chestakova, Anne van der Linden, Marjan Boter, Emmanuelle Munger, Corine GeurtsvanKessel, Annemiek van der Eijk, Richard Molenkamp, Marion Koopmans, on behalf of the Dutch national COVID-19 response team. |
| EPI_ISL_1311430 | Dutch COVID-19 response team | Erasmus Medical Centre | Bas Oude Munnink, Reina Sikkema, David Nieuwenhuijse, Irina Chestakova, Anne van der Linden, Marjan Boter, Emmanuelle Munger, Corine GeurtsvanKessel, Annemiek van der Eijk, Richard Molenkamp, Marion Koopmans, on behalf of the Dutch national COVID-19 response team. |
| EPI_ISL_1311433 | Dutch COVID-19 response team | Erasmus Medical Centre | Bas Oude Munnink, Reina Sikkema, David Nieuwenhuijse, Irina Chestakova, Anne van der Linden, Marjan Boter, Emmanuelle Munger, Corine GeurtsvanKessel, Annemiek van der Eijk, Richard Molenkamp, Marion Koopmans, on behalf of the Dutch national COVID-19 response team. |
| EPI_ISL_1311608 | Dutch COVID-19 response team | Erasmus Medical Centre | Bas Oude Munnink, Reina Sikkema, David Nieuwenhuijse, Irina Chestakova, Anne van der Linden, Marjan Boter, Emmanuelle Munger, Corine GeurtsvanKessel, Annemiek van der Eijk, Richard Molenkamp, Marion Koopmans, on behalf of the Dutch national COVID-19 response team. |
| EPI_ISL_1311310 | Dutch COVID-19 response team | Erasmus Medical Centre | Bas Oude Munnink, Reina Sikkema, David Nieuwenhuijse, Irina Chestakova, Anne van der Linden, Marjan Boter, Emmanuelle Munger, Corine GeurtsvanKessel, Annemiek van der Eijk, Richard Molenkamp, Marion Koopmans, on behalf of the Dutch national COVID-19 response team. |
| EPI_ISL_1311470 | Dutch COVID-19 response team | Erasmus Medical Centre | Bas Oude Munnink, Reina Sikkema, David Nieuwenhuijse, Irina Chestakova, Anne van der Linden, Marjan Boter, Emmanuelle Munger, Corine GeurtsvanKessel, Annemiek van der Eijk, Richard Molenkamp, Marion Koopmans, on behalf of the Dutch national COVID-19 response team. |
| EPI_ISL_1311474 | Dutch COVID-19 response team | Erasmus Medical Centre | Bas Oude Munnink, Reina Sikkema, David Nieuwenhuijse, Irina Chestakova, Anne van der Linden, Marjan Boter, Emmanuelle Munger, Corine GeurtsvanKessel, Annemiek van der Eijk, Richard Molenkamp, Marion Koopmans, on behalf of the Dutch national COVID-19 response team. |
| EPI_ISL_1311665 | Dutch COVID-19 response team | Erasmus Medical Centre | Bas Oude Munnink, Reina Sikkema, David Nieuwenhuijse, Irina Chestakova, Anne van der Linden, Marjan Boter, Emmanuelle Munger, Corine GeurtsvanKessel, Annemiek van der Eijk, Richard Molenkamp, Marion Koopmans, on behalf of the Dutch national COVID-19 response team. |
| EPI_ISL_1311680 | Dutch COVID-19 response team | Erasmus Medical Centre | Bas Oude Munnink, Reina Sikkema, David Nieuwenhuijse, Irina Chestakova, Anne van der Linden, Marjan Boter, Emmanuelle Munger, Corine GeurtsvanKessel, Annemiek van der Eijk, Richard Molenkamp, Marion Koopmans, on behalf of the Dutch national COVID-19 response team. |
| EPI_ISL_1311731 | Dutch COVID-19 response team | Erasmus Medical Centre | Bas Oude Munnink, Reina Sikkema, David Nieuwenhuijse, Irina Chestakova, Anne van der Linden, Marjan Boter, Emmanuelle Munger, Corine GeurtsvanKessel, Annemiek van der Eijk, Richard Molenkamp, Marion Koopmans, on behalf of the Dutch national COVID-19 response team. |
| EPI_ISL_1311742 | Dutch COVID-19 response team | Erasmus Medical Centre | Bas Oude Munnink, Reina Sikkema, David Nieuwenhuijse, Irina Chestakova, Anne van der Linden, Marjan Boter, Emmanuelle Munger, Corine GeurtsvanKessel, Annemiek van der Eijk, Richard Molenkamp, Marion Koopmans, on behalf of the Dutch national COVID-19 response team. |
| EPI_ISL_965808 | Dutch COVID-19 response team | Medical Microbiology, Maastricht University Medical Centre | Jozef Dingemans*, Brian van der Veer*, Erik Beuken, Carmen Reumkens, Lieke van Alphen, Christian Hoebe, Paul Savelkoul |
| EPI_ISL_1299281 | Microvida | Microvida | S.D. Pas, J.J. Verweij, J. Stohr |
| EPI_ISL_905542 | Dutch COVID-19 response team | National Institute for Public Health and the Environment (RIVM) | Adam Meijer, Harry Vennema, Dirk Eggink, Jeroen Cremer, Sharon van den Brink, Bas van der Veer, AnneMarie van den Brandt, Florian Zwagemaker, Dennis Schmitz, Chantal Reusken, on behalf of the national COVID-19 response team |
| EPI_ISL_905270 | Dutch COVID-19 response team | National Institute for Public Health and the Environment (RIVM) | Adam Meijer, Harry Vennema, Dirk Eggink, Jeroen Cremer, Sharon van den Brink, Bas van der Veer, AnneMarie van den Brandt, Florian Zwagemaker, Dennis Schmitz, Chantal Reusken, on behalf of the national COVID-19 response team |
| EPI_ISL_905271 | Dutch COVID-19 response team | National Institute for Public Health and the Environment (RIVM) | Adam Meijer, Harry Vennema, Dirk Eggink, Jeroen Cremer, Sharon van den Brink, Bas van der Veer, AnneMarie van den Brandt, Florian Zwagemaker, Dennis Schmitz, Chantal Reusken, on behalf of the national COVID-19 response team |
| EPI_ISL_905280 | Dutch COVID-19 response team | National Institute for Public Health and the Environment (RIVM) | Adam Meijer, Harry Vennema, Dirk Eggink, Jeroen Cremer, Sharon van den Brink, Bas van der Veer, AnneMarie van den Brandt, Florian Zwagemaker, Dennis Schmitz, Chantal Reusken, on behalf of the national COVID-19 response team |
| EPI_ISL_905281 | Dutch COVID-19 response team | National Institute for Public Health and the Environment (RIVM) | Adam Meijer, Harry Vennema, Dirk Eggink, Jeroen Cremer, Sharon van den Brink, Bas van der Veer, AnneMarie van den Brandt, Florian Zwagemaker, Dennis Schmitz, Chantal Reusken, on behalf of the national COVID-19 response team |
| EPI_ISL_905291 | Dutch COVID-19 response team | National Institute for Public Health and the Environment (RIVM) | Adam Meijer, Harry Vennema, Dirk Eggink, Jeroen Cremer, Sharon van den Brink, Bas van der Veer, AnneMarie van den Brandt, Florian Zwagemaker, Dennis Schmitz, Chantal Reusken, on behalf of the national COVID-19 response team |
| EPI_ISL_904815 | Dutch COVID-19 response team | National Institute for Public Health and the Environment (RIVM) | Adam Meijer, Harry Vennema, Dirk Eggink, Jeroen Cremer, Sharon van den Brink, Bas van der Veer, AnneMarie van den Brandt, Florian Zwagemaker, Dennis Schmitz, Chantal Reusken, on behalf of the national COVID-19 response team |
| EPI_ISL_904722 | Dutch COVID-19 response team | National Institute for Public Health and the Environment (RIVM) | Adam Meijer, Harry Vennema, Dirk Eggink, Jeroen Cremer, Sharon van den Brink, Bas van der Veer, AnneMarie van den Brandt, Florian Zwagemaker, Dennis Schmitz, Chantal Reusken, on behalf of the national COVID-19 response team |
| EPI_ISL_904824 | Dutch COVID-19 response team | National Institute for Public Health and the Environment (RIVM) | Adam Meijer, Harry Vennema, Dirk Eggink, Jeroen Cremer, Sharon van den Brink, Bas van der Veer, AnneMarie van den Brandt, Florian Zwagemaker, Dennis Schmitz, Chantal Reusken, on behalf of the national COVID-19 response team |
| EPI_ISL_904826 | Dutch COVID-19 response team | National Institute for Public Health and the Environment (RIVM) | Adam Meijer, Harry Vennema, Dirk Eggink, Jeroen Cremer, Sharon van den Brink, Bas van der Veer, AnneMarie van den Brandt, Florian Zwagemaker, Dennis Schmitz, Chantal Reusken, on behalf of the national COVID-19 response team |
| EPI_ISL_904834 | Dutch COVID-19 response team | National Institute for Public Health and the Environment (RIVM) | Adam Meijer, Harry Vennema, Dirk Eggink, Jeroen Cremer, Sharon van den Brink, Bas van der Veer, AnneMarie van den Brandt, Florian Zwagemaker, Dennis Schmitz, Chantal Reusken, on behalf of the national COVID-19 response team |
| EPI_ISL_904835 | Dutch COVID-19 response team | National Institute for Public Health and the Environment (RIVM) | Adam Meijer, Harry Vennema, Dirk Eggink, Jeroen Cremer, Sharon van den Brink, Bas van der Veer, AnneMarie van den Brandt, Florian Zwagemaker, Dennis Schmitz, Chantal Reusken, on behalf of the national COVID-19 response team |
| EPI_ISL_904840 | Dutch COVID-19 response team | National Institute for Public Health and the Environment (RIVM) | Adam Meijer, Harry Vennema, Dirk Eggink, Jeroen Cremer, Sharon van den Brink, Bas van der Veer, AnneMarie van den Brandt, Florian Zwagemaker, Dennis Schmitz, Chantal Reusken, on behalf of the national COVID-19 response team |
| EPI_ISL_943149 | Dutch COVID-19 response team | National Institute for Public Health and the Environment (RIVM) | Adam Meijer, Harry Vennema, Dirk Eggink, Jeroen Cremer, Sharon van den Brink, Bas van der Veer, AnneMarie van den Brandt, Florian Zwagemaker, Dennis Schmitz, Chantal Reusken, on behalf of the national COVID-19 response team |
| EPI_ISL_943205 | Dutch COVID-19 response team | National Institute for Public Health and the Environment (RIVM) | Adam Meijer, Harry Vennema, Dirk Eggink, Jeroen Cremer, Sharon van den Brink, Bas van der Veer, AnneMarie van den Brandt, Florian Zwagemaker, Dennis Schmitz, Chantal Reusken, on behalf of the national COVID-19 response team |
| EPI_ISL_943349 | Dutch COVID-19 response team | National Institute for Public Health and the Environment (RIVM) | Adam Meijer, Harry Vennema, Dirk Eggink, Jeroen Cremer, Sharon van den Brink, Bas van der Veer, AnneMarie van den Brandt, Florian Zwagemaker, Dennis Schmitz, Chantal Reusken, on behalf of the national COVID-19 response team |
| EPI_ISL_943354 | Dutch COVID-19 response team | National Institute for Public Health and the Environment (RIVM) | Adam Meijer, Harry Vennema, Dirk Eggink, Jeroen Cremer, Sharon van den Brink, Bas van der Veer, AnneMarie van den Brandt, Florian Zwagemaker, Dennis Schmitz, Chantal Reusken, on behalf of the national COVID-19 response team |
| EPI_ISL_943363 | Dutch COVID-19 response team | National Institute for Public Health and the Environment (RIVM) | Adam Meijer, Harry Vennema, Dirk Eggink, Jeroen Cremer, Sharon van den Brink, Bas van der Veer, AnneMarie van den Brandt, Florian Zwagemaker, Dennis Schmitz, Chantal Reusken, on behalf of the national COVID-19 response team |
| EPI_ISL_943370 | Dutch COVID-19 response team | National Institute for Public Health and the Environment (RIVM) | Adam Meijer, Harry Vennema, Dirk Eggink, Jeroen Cremer, Sharon van den Brink, Bas van der Veer, AnneMarie van den Brandt, Florian Zwagemaker, Dennis Schmitz, Chantal Reusken, on behalf of the national COVID-19 response team |
| EPI_ISL_943378 | Dutch COVID-19 response team | National Institute for Public Health and the Environment (RIVM) | Adam Meijer, Harry Vennema, Dirk Eggink, Jeroen Cremer, Sharon van den Brink, Bas van der Veer, AnneMarie van den Brandt, Florian Zwagemaker, Dennis Schmitz, Chantal Reusken, on behalf of the national COVID-19 response team |
| EPI_ISL_943392 | Dutch COVID-19 response team | National Institute for Public Health and the Environment (RIVM) | Adam Meijer, Harry Vennema, Dirk Eggink, Jeroen Cremer, Sharon van den Brink, Bas van der Veer, AnneMarie van den Brandt, Florian Zwagemaker, Dennis Schmitz, Chantal Reusken, on behalf of the national COVID-19 response team |
| EPI_ISL_943394 | Dutch COVID-19 response team | National Institute for Public Health and the Environment (RIVM) | Adam Meijer, Harry Vennema, Dirk Eggink, Jeroen Cremer, Sharon van den Brink, Bas van der Veer, AnneMarie van den Brandt, Florian Zwagemaker, Dennis Schmitz, Chantal Reusken, on behalf of the national COVID-19 response team |
| EPI_ISL_943396 | Dutch COVID-19 response team | National Institute for Public Health and the Environment (RIVM) | Adam Meijer, Harry Vennema, Dirk Eggink, Jeroen Cremer, Sharon van den Brink, Bas van der Veer, AnneMarie van den Brandt, Florian Zwagemaker, Dennis Schmitz, Chantal Reusken, on behalf of the national COVID-19 response team |
| EPI_ISL_943402 | Dutch COVID-19 response team | National Institute for Public Health and the Environment (RIVM) | Adam Meijer, Harry Vennema, Dirk Eggink, Jeroen Cremer, Sharon van den Brink, Bas van der Veer, AnneMarie van den Brandt, Florian Zwagemaker, Dennis Schmitz, Chantal Reusken, on behalf of the national COVID-19 response team |
| EPI_ISL_943403 | Dutch COVID-19 response team | National Institute for Public Health and the Environment (RIVM) | Adam Meijer, Harry Vennema, Dirk Eggink, Jeroen Cremer, Sharon van den Brink, Bas van der Veer, AnneMarie van den Brandt, Florian Zwagemaker, Dennis Schmitz, Chantal Reusken, on behalf of the national COVID-19 response team |
| EPI_ISL_943412 | Dutch COVID-19 response team | National Institute for Public Health and the Environment (RIVM) | Adam Meijer, Harry Vennema, Dirk Eggink, Jeroen Cremer, Sharon van den Brink, Bas van der Veer, AnneMarie van den Brandt, Florian Zwagemaker, Dennis Schmitz, Chantal Reusken, on behalf of the national COVID-19 response team |
| EPI_ISL_1013916 | Dutch COVID-19 response team | National Institute for Public Health and the Environment (RIVM) | Adam Meijer, Harry Vennema, Dirk Eggink, Jeroen Cremer, Sharon van den Brink, Bas van der Veer, AnneMarie van den Brandt, Florian Zwagemaker, Dennis Schmitz, Chantal Reusken, on behalf of the national COVID-19 response team |
| EPI_ISL_1013918 | Dutch COVID-19 response team | National Institute for Public Health and the Environment (RIVM) | Adam Meijer, Harry Vennema, Dirk Eggink, Jeroen Cremer, Sharon van den Brink, Bas van der Veer, AnneMarie van den Brandt, Florian Zwagemaker, Dennis Schmitz, Chantal Reusken, on behalf of the national COVID-19 response team |
| EPI_ISL_1013924 | Dutch COVID-19 response team | National Institute for Public Health and the Environment (RIVM) | Adam Meijer, Harry Vennema, Dirk Eggink, Jeroen Cremer, Sharon van den Brink, Bas van der Veer, AnneMarie van den Brandt, Florian Zwagemaker, Dennis Schmitz, Chantal Reusken, on behalf of the national COVID-19 response team |
| EPI_ISL_1035651 | Dutch COVID-19 response team | National Institute for Public Health and the Environment (RIVM) | Adam Meijer, Harry Vennema, Dirk Eggink, Jeroen Cremer, Sharon van den Brink, Bas van der Veer, AnneMarie van den Brandt, Florian Zwagemaker, Dennis Schmitz, Chantal Reusken, on behalf of the national COVID-19 response team |
| EPI_ISL_1035624 | Dutch COVID-19 response team | National Institute for Public Health and the Environment (RIVM) | Adam Meijer, Harry Vennema, Dirk Eggink, Jeroen Cremer, Sharon van den Brink, Bas van der Veer, AnneMarie van den Brandt, Florian Zwagemaker, Dennis Schmitz, Chantal Reusken, on behalf of the national COVID-19 response team |
| EPI_ISL_1035619 | Dutch COVID-19 response team | National Institute for Public Health and the Environment (RIVM) | Adam Meijer, Harry Vennema, Dirk Eggink, Jeroen Cremer, Sharon van den Brink, Bas van der Veer, AnneMarie van den Brandt, Florian Zwagemaker, Dennis Schmitz, Chantal Reusken, on behalf of the national COVID-19 response team |
| EPI_ISL_1035608 | Dutch COVID-19 response team | National Institute for Public Health and the Environment (RIVM) | Adam Meijer, Harry Vennema, Dirk Eggink, Jeroen Cremer, Sharon van den Brink, Bas van der Veer, AnneMarie van den Brandt, Florian Zwagemaker, Dennis Schmitz, Chantal Reusken, on behalf of the national COVID-19 response team |
| EPI_ISL_1089963 | Dutch COVID-19 response team | National Institute for Public Health and the Environment (RIVM) | Adam Meijer, Harry Vennema, Dirk Eggink, Jeroen Cremer, Sharon van den Brink, Bas van der Veer, AnneMarie van den Brandt, Florian Zwagemaker, Dennis Schmitz, Chantal Reusken, on behalf of the national COVID-19 response team |
| EPI_ISL_1089882 | Dutch COVID-19 response team | National Institute for Public Health and the Environment (RIVM) | Adam Meijer, Harry Vennema, Dirk Eggink, Jeroen Cremer, Sharon van den Brink, Bas van der Veer, AnneMarie van den Brandt, Florian Zwagemaker, Dennis Schmitz, Chantal Reusken, on behalf of the national COVID-19 response team |
| EPI_ISL_1090053 | Dutch COVID-19 response team | National Institute for Public Health and the Environment (RIVM) | Adam Meijer, Harry Vennema, Dirk Eggink, Jeroen Cremer, Sharon van den Brink, Bas van der Veer, AnneMarie van den Brandt, Florian Zwagemaker, Dennis Schmitz, Chantal Reusken, on behalf of the national COVID-19 response team |
| EPI_ISL_1090106 | Dutch COVID-19 response team | National Institute for Public Health and the Environment (RIVM) | Adam Meijer, Harry Vennema, Dirk Eggink, Jeroen Cremer, Sharon van den Brink, Bas van der Veer, AnneMarie van den Brandt, Florian Zwagemaker, Dennis Schmitz, Chantal Reusken, on behalf of the national COVID-19 response team |
| EPI_ISL_1089939 | Dutch COVID-19 response team | National Institute for Public Health and the Environment (RIVM) | Adam Meijer, Harry Vennema, Dirk Eggink, Jeroen Cremer, Sharon van den Brink, Bas van der Veer, AnneMarie van den Brandt, Florian Zwagemaker, Dennis Schmitz, Chantal Reusken, on behalf of the national COVID-19 response team |
| EPI_ISL_1089953 | Dutch COVID-19 response team | National Institute for Public Health and the Environment (RIVM) | Adam Meijer, Harry Vennema, Dirk Eggink, Jeroen Cremer, Sharon van den Brink, Bas van der Veer, AnneMarie van den Brandt, Florian Zwagemaker, Dennis Schmitz, Chantal Reusken, on behalf of the national COVID-19 response team |
| EPI_ISL_1090003 | Dutch COVID-19 response team | National Institute for Public Health and the Environment (RIVM) | Adam Meijer, Harry Vennema, Dirk Eggink, Jeroen Cremer, Sharon van den Brink, Bas van der Veer, AnneMarie van den Brandt, Florian Zwagemaker, Dennis Schmitz, Chantal Reusken, on behalf of the national COVID-19 response team |
| EPI_ISL_1089780 | Dutch COVID-19 response team | National Institute for Public Health and the Environment (RIVM) | Adam Meijer, Harry Vennema, Dirk Eggink, Jeroen Cremer, Sharon van den Brink, Bas van der Veer, AnneMarie van den Brandt, Florian Zwagemaker, Dennis Schmitz, Chantal Reusken, on behalf of the national COVID-19 response team |
| EPI_ISL_1090238 | Dutch COVID-19 response team | National Institute for Public Health and the Environment (RIVM) | Adam Meijer, Harry Vennema, Dirk Eggink, Jeroen Cremer, Sharon van den Brink, Bas van der Veer, AnneMarie van den Brandt, Florian Zwagemaker, Dennis Schmitz, Chantal Reusken, on behalf of the national COVID-19 response team |
| EPI_ISL_1090185 | Dutch COVID-19 response team | National Institute for Public Health and the Environment (RIVM) | Adam Meijer, Harry Vennema, Dirk Eggink, Jeroen Cremer, Sharon van den Brink, Bas van der Veer, AnneMarie van den Brandt, Florian Zwagemaker, Dennis Schmitz, Chantal Reusken, on behalf of the national COVID-19 response team |
| EPI_ISL_1090226 | Dutch COVID-19 response team | National Institute for Public Health and the Environment (RIVM) | Adam Meijer, Harry Vennema, Dirk Eggink, Jeroen Cremer, Sharon van den Brink, Bas van der Veer, AnneMarie van den Brandt, Florian Zwagemaker, Dennis Schmitz, Chantal Reusken, on behalf of the national COVID-19 response team |
| EPI_ISL_1090092 | Dutch COVID-19 response team | National Institute for Public Health and the Environment (RIVM) | Adam Meijer, Harry Vennema, Dirk Eggink, Jeroen Cremer, Sharon van den Brink, Bas van der Veer, AnneMarie van den Brandt, Florian Zwagemaker, Dennis Schmitz, Chantal Reusken, on behalf of the national COVID-19 response team |
| EPI_ISL_1090147 | Dutch COVID-19 response team | National Institute for Public Health and the Environment (RIVM) | Adam Meijer, Harry Vennema, Dirk Eggink, Jeroen Cremer, Sharon van den Brink, Bas van der Veer, AnneMarie van den Brandt, Florian Zwagemaker, Dennis Schmitz, Chantal Reusken, on behalf of the national COVID-19 response team |
| EPI_ISL_1090252 | Dutch COVID-19 response team | National Institute for Public Health and the Environment (RIVM) | Adam Meijer, Harry Vennema, Dirk Eggink, Jeroen Cremer, Sharon van den Brink, Bas van der Veer, AnneMarie van den Brandt, Florian Zwagemaker, Dennis Schmitz, Chantal Reusken, on behalf of the national COVID-19 response team |
| EPI_ISL_1165721 | Dutch COVID-19 response team | National Institute for Public Health and the Environment (RIVM) | Adam Meijer, Harry Vennema, Dirk Eggink, Jeroen Cremer, Sharon van den Brink, Bas van der Veer, AnneMarie van den Brandt, Florian Zwagemaker, Dennis Schmitz, Chantal Reusken, on behalf of the national COVID-19 response team |
| EPI_ISL_1165723 | Dutch COVID-19 response team | National Institute for Public Health and the Environment (RIVM) | Adam Meijer, Harry Vennema, Dirk Eggink, Jeroen Cremer, Sharon van den Brink, Bas van der Veer, AnneMarie van den Brandt, Florian Zwagemaker, Dennis Schmitz, Chantal Reusken, on behalf of the national COVID-19 response team |
| EPI_ISL_1165725 | Dutch COVID-19 response team | National Institute for Public Health and the Environment (RIVM) | Adam Meijer, Harry Vennema, Dirk Eggink, Jeroen Cremer, Sharon van den Brink, Bas van der Veer, AnneMarie van den Brandt, Florian Zwagemaker, Dennis Schmitz, Chantal Reusken, on behalf of the national COVID-19 response team |
| EPI_ISL_1165727 | Dutch COVID-19 response team | National Institute for Public Health and the Environment (RIVM) | Adam Meijer, Harry Vennema, Dirk Eggink, Jeroen Cremer, Sharon van den Brink, Bas van der Veer, AnneMarie van den Brandt, Florian Zwagemaker, Dennis Schmitz, Chantal Reusken, on behalf of the national COVID-19 response team |
| EPI_ISL_1165728 | Dutch COVID-19 response team | National Institute for Public Health and the Environment (RIVM) | Adam Meijer, Harry Vennema, Dirk Eggink, Jeroen Cremer, Sharon van den Brink, Bas van der Veer, AnneMarie van den Brandt, Florian Zwagemaker, Dennis Schmitz, Chantal Reusken, on behalf of the national COVID-19 response team |
| EPI_ISL_1165743 | Dutch COVID-19 response team | National Institute for Public Health and the Environment (RIVM) | Adam Meijer, Harry Vennema, Dirk Eggink, Jeroen Cremer, Sharon van den Brink, Bas van der Veer, AnneMarie van den Brandt, Florian Zwagemaker, Dennis Schmitz, Chantal Reusken, on behalf of the national COVID-19 response team |
| EPI_ISL_1165745 | Dutch COVID-19 response team | National Institute for Public Health and the Environment (RIVM) | Adam Meijer, Harry Vennema, Dirk Eggink, Jeroen Cremer, Sharon van den Brink, Bas van der Veer, AnneMarie van den Brandt, Florian Zwagemaker, Dennis Schmitz, Chantal Reusken, on behalf of the national COVID-19 response team |
| EPI_ISL_1216308 | Dutch COVID-19 response team | National Institute for Public Health and the Environment (RIVM) | Adam Meijer, Harry Vennema, Dirk Eggink, Jeroen Cremer, Sharon van den Brink, Bas van der Veer, AnneMarie van den Brandt, Florian Zwagemaker, Dennis Schmitz, Chantal Reusken, on behalf of the national COVID-19 response team |
| EPI_ISL_1232383 | Dutch COVID-19 response team | National Institute for Public Health and the Environment (RIVM) | Adam Meijer, Harry Vennema, Dirk Eggink, Jeroen Cremer, Sharon van den Brink, Bas van der Veer, AnneMarie van den Brandt, Florian Zwagemaker, Dennis Schmitz, Chantal Reusken, on behalf of the national COVID-19 response team |
| EPI_ISL_1232969 | Dutch COVID-19 response team | National Institute for Public Health and the Environment (RIVM) | Adam Meijer, Harry Vennema, Dirk Eggink, Jeroen Cremer, Sharon van den Brink, Bas van der Veer, AnneMarie van den Brandt, Florian Zwagemaker, Dennis Schmitz, Chantal Reusken, on behalf of the national COVID-19 response team |
| EPI_ISL_1232988 | Dutch COVID-19 response team | National Institute for Public Health and the Environment (RIVM) | Adam Meijer, Harry Vennema, Dirk Eggink, Jeroen Cremer, Sharon van den Brink, Bas van der Veer, AnneMarie van den Brandt, Florian Zwagemaker, Dennis Schmitz, Chantal Reusken, on behalf of the national COVID-19 response team |
| EPI_ISL_1232991 | Dutch COVID-19 response team | National Institute for Public Health and the Environment (RIVM) | Adam Meijer, Harry Vennema, Dirk Eggink, Jeroen Cremer, Sharon van den Brink, Bas van der Veer, AnneMarie van den Brandt, Florian Zwagemaker, Dennis Schmitz, Chantal Reusken, on behalf of the national COVID-19 response team |
| EPI_ISL_1233004 | Dutch COVID-19 response team | National Institute for Public Health and the Environment (RIVM) | Adam Meijer, Harry Vennema, Dirk Eggink, Jeroen Cremer, Sharon van den Brink, Bas van der Veer, AnneMarie van den Brandt, Florian Zwagemaker, Dennis Schmitz, Chantal Reusken, on behalf of the national COVID-19 response team |
| EPI_ISL_1289305 | Dutch COVID-19 response team | National Institute for Public Health and the Environment (RIVM) | Adam Meijer, Harry Vennema, Dirk Eggink, Jeroen Cremer, Sharon van den Brink, Bas van der Veer, AnneMarie van den Brandt, Florian Zwagemaker, Dennis Schmitz, Chantal Reusken, on behalf of the national COVID-19 response team |
| EPI_ISL_1371442 | Dutch COVID-19 response team | National Institute for Public Health and the Environment (RIVM) | Adam Meijer, Harry Vennema, Dirk Eggink, Jeroen Cremer, Sharon van den Brink, Bas van der Veer, AnneMarie van den Brandt, Florian Zwagemaker, Dennis Schmitz, Chantal Reusken, on behalf of the national COVID-19 response team |
| EPI_ISL_1371541 | Dutch COVID-19 response team | National Institute for Public Health and the Environment (RIVM) | Adam Meijer, Harry Vennema, Dirk Eggink, Jeroen Cremer, Sharon van den Brink, Bas van der Veer, AnneMarie van den Brandt, Florian Zwagemaker, Dennis Schmitz, Chantal Reusken, on behalf of the national COVID-19 response team |
| EPI_ISL_1370687 | Dutch COVID-19 response team | National Institute for Public Health and the Environment (RIVM) | Adam Meijer, Harry Vennema, Dirk Eggink, Jeroen Cremer, Sharon van den Brink, Bas van der Veer, AnneMarie van den Brandt, Florian Zwagemaker, Dennis Schmitz, Chantal Reusken, on behalf of the national COVID-19 response team |
| EPI_ISL_1370837 | Dutch COVID-19 response team | National Institute for Public Health and the Environment (RIVM) | Adam Meijer, Harry Vennema, Dirk Eggink, Jeroen Cremer, Sharon van den Brink, Bas van der Veer, AnneMarie van den Brandt, Florian Zwagemaker, Dennis Schmitz, Chantal Reusken, on behalf of the national COVID-19 response team |
| EPI_ISL_723363 | Dutch COVID-19 response team | National Institute for Public Health and the Environment (RIVM) | Adam Meijer, Harry Vennema, Dirk Eggink, Jeroen Cremer, Sharon van den Brink, Bas van der Veer, AnneMarie van den Brandt, Florian Zwagemaker, Dennis Schmitz, Chantal Reusken, on behalf of the national COVID-19 response team |
| EPI_ISL_723383 | Dutch COVID-19 response team | National Institute for Public Health and the Environment (RIVM) | Adam Meijer, Harry Vennema, Dirk Eggink, Jeroen Cremer, Sharon van den Brink, Bas van der Veer, AnneMarie van den Brandt, Florian Zwagemaker, Dennis Schmitz, Chantal Reusken, on behalf of the national COVID-19 response team |
| EPI_ISL_723409 | Dutch COVID-19 response team | National Institute for Public Health and the Environment (RIVM) | Adam Meijer, Harry Vennema, Dirk Eggink, Jeroen Cremer, Sharon van den Brink, Bas van der Veer, AnneMarie van den Brandt, Florian Zwagemaker, Dennis Schmitz, Chantal Reusken, on behalf of the national COVID-19 response team |
| EPI_ISL_723157 | Dutch COVID-19 response team | National Institute for Public Health and the Environment (RIVM) | Adam Meijer, Harry Vennema, Dirk Eggink, Jeroen Cremer, Sharon van den Brink, Bas van der Veer, AnneMarie van den Brandt, Florian Zwagemaker, Dennis Schmitz, Chantal Reusken, on behalf of the national COVID-19 response team |
| EPI_ISL_723417 | Dutch COVID-19 response team | National Institute for Public Health and the Environment (RIVM) | Adam Meijer, Harry Vennema, Dirk Eggink, Jeroen Cremer, Sharon van den Brink, Bas van der Veer, AnneMarie van den Brandt, Florian Zwagemaker, Dennis Schmitz, Chantal Reusken, on behalf of the national COVID-19 response team |
| EPI_ISL_723419 | Dutch COVID-19 response team | National Institute for Public Health and the Environment (RIVM) | Adam Meijer, Harry Vennema, Dirk Eggink, Jeroen Cremer, Sharon van den Brink, Bas van der Veer, AnneMarie van den Brandt, Florian Zwagemaker, Dennis Schmitz, Chantal Reusken, on behalf of the national COVID-19 response team |
| EPI_ISL_723421 | Dutch COVID-19 response team | National Institute for Public Health and the Environment (RIVM) | Adam Meijer, Harry Vennema, Dirk Eggink, Jeroen Cremer, Sharon van den Brink, Bas van der Veer, AnneMarie van den Brandt, Florian Zwagemaker, Dennis Schmitz, Chantal Reusken, on behalf of the national COVID-19 response team |
| EPI_ISL_728571 | Dutch COVID-19 response team | National Institute for Public Health and the Environment (RIVM) | Adam Meijer, Harry Vennema, Dirk Eggink, Jeroen Cremer, Sharon van den Brink, Bas van der Veer, AnneMarie van den Brandt, Florian Zwagemaker, Dennis Schmitz, Chantal Reusken, on behalf of the national COVID-19 response team |
| EPI_ISL_728572 | Dutch COVID-19 response team | National Institute for Public Health and the Environment (RIVM) | Adam Meijer, Harry Vennema, Dirk Eggink, Jeroen Cremer, Sharon van den Brink, Bas van der Veer, AnneMarie van den Brandt, Florian Zwagemaker, Dennis Schmitz, Chantal Reusken, on behalf of the national COVID-19 response team |
| EPI_ISL_728573 | Dutch COVID-19 response team | National Institute for Public Health and the Environment (RIVM) | Adam Meijer, Harry Vennema, Dirk Eggink, Jeroen Cremer, Sharon van den Brink, Bas van der Veer, AnneMarie van den Brandt, Florian Zwagemaker, Dennis Schmitz, Chantal Reusken, on behalf of the national COVID-19 response team |
| EPI_ISL_728574 | Dutch COVID-19 response team | National Institute for Public Health and the Environment (RIVM) | Adam Meijer, Harry Vennema, Dirk Eggink, Jeroen Cremer, Sharon van den Brink, Bas van der Veer, AnneMarie van den Brandt, Florian Zwagemaker, Dennis Schmitz, Chantal Reusken, on behalf of the national COVID-19 response team |
| EPI_ISL_728576 | Dutch COVID-19 response team | National Institute for Public Health and the Environment (RIVM) | Adam Meijer, Harry Vennema, Dirk Eggink, Jeroen Cremer, Sharon van den Brink, Bas van der Veer, AnneMarie van den Brandt, Florian Zwagemaker, Dennis Schmitz, Chantal Reusken, on behalf of the national COVID-19 response team |
| EPI_ISL_1457544 | Dutch COVID-19 response team | National Institute for Public Health and the Environment (RIVM) | Adam Meijer, Harry Vennema, Dirk Eggink, Jeroen Cremer, Sharon van den Brink, Bas van der Veer, AnneMarie van den Brandt, Florian Zwagemaker, Dennis Schmitz, Chantal Reusken, on behalf of the national COVID-19 response team |
| EPI_ISL_728745 | Dutch COVID-19 response team | National Institute for Public Health and the Environment (RIVM) | Adam Meijer, Harry Vennema, Dirk Eggink, Jeroen Cremer, Sharon van den Brink, Bas van der Veer, AnneMarie van den Brandt, Florian Zwagemaker, Dennis Schmitz, Chantal Reusken, on behalf of the national COVID-19 response team |
| EPI_ISL_790657 | Dutch COVID-19 response team | National Institute for Public Health and the Environment (RIVM) | Adam Meijer, Harry Vennema, Dirk Eggink, Jeroen Cremer, Sharon van den Brink, Bas van der Veer, AnneMarie van den Brandt, Florian Zwagemaker, Dennis Schmitz, Chantal Reusken, on behalf of the national COVID-19 response team |
| EPI_ISL_790660 | Dutch COVID-19 response team | National Institute for Public Health and the Environment (RIVM) | Adam Meijer, Harry Vennema, Dirk Eggink, Jeroen Cremer, Sharon van den Brink, Bas van der Veer, AnneMarie van den Brandt, Florian Zwagemaker, Dennis Schmitz, Chantal Reusken, on behalf of the national COVID-19 response team |
| EPI_ISL_1457738 | Dutch COVID-19 response team | National Institute for Public Health and the Environment (RIVM) | Adam Meijer, Harry Vennema, Dirk Eggink, Jeroen Cremer, Sharon van den Brink, Bas van der Veer, AnneMarie van den Brandt, Florian Zwagemaker, Dennis Schmitz, Chantal Reusken, on behalf of the national COVID-19 response team |
| EPI_ISL_790688 | Dutch COVID-19 response team | National Institute for Public Health and the Environment (RIVM) | Adam Meijer, Harry Vennema, Dirk Eggink, Jeroen Cremer, Sharon van den Brink, Bas van der Veer, AnneMarie van den Brandt, Florian Zwagemaker, Dennis Schmitz, Chantal Reusken, on behalf of the national COVID-19 response team |
| EPI_ISL_790689 | Dutch COVID-19 response team | National Institute for Public Health and the Environment (RIVM) | Adam Meijer, Harry Vennema, Dirk Eggink, Jeroen Cremer, Sharon van den Brink, Bas van der Veer, AnneMarie van den Brandt, Florian Zwagemaker, Dennis Schmitz, Chantal Reusken, on behalf of the national COVID-19 response team |
| EPI_ISL_790690 | Dutch COVID-19 response team | National Institute for Public Health and the Environment (RIVM) | Adam Meijer, Harry Vennema, Dirk Eggink, Jeroen Cremer, Sharon van den Brink, Bas van der Veer, AnneMarie van den Brandt, Florian Zwagemaker, Dennis Schmitz, Chantal Reusken, on behalf of the national COVID-19 response team |
| EPI_ISL_790691 | Dutch COVID-19 response team | National Institute for Public Health and the Environment (RIVM) | Adam Meijer, Harry Vennema, Dirk Eggink, Jeroen Cremer, Sharon van den Brink, Bas van der Veer, AnneMarie van den Brandt, Florian Zwagemaker, Dennis Schmitz, Chantal Reusken, on behalf of the national COVID-19 response team |
| EPI_ISL_790592 | Dutch COVID-19 response team | National Institute for Public Health and the Environment (RIVM) | Adam Meijer, Harry Vennema, Dirk Eggink, Jeroen Cremer, Sharon van den Brink, Bas van der Veer, AnneMarie van den Brandt, Florian Zwagemaker, Dennis Schmitz, Chantal Reusken, on behalf of the national COVID-19 response team |
| EPI_ISL_790730 | Dutch COVID-19 response team | National Institute for Public Health and the Environment (RIVM) | Adam Meijer, Harry Vennema, Dirk Eggink, Jeroen Cremer, Sharon van den Brink, Bas van der Veer, AnneMarie van den Brandt, Florian Zwagemaker, Dennis Schmitz, Chantal Reusken, on behalf of the national COVID-19 response team |
| EPI_ISL_790731 | Dutch COVID-19 response team | National Institute for Public Health and the Environment (RIVM) | Adam Meijer, Harry Vennema, Dirk Eggink, Jeroen Cremer, Sharon van den Brink, Bas van der Veer, AnneMarie van den Brandt, Florian Zwagemaker, Dennis Schmitz, Chantal Reusken, on behalf of the national COVID-19 response team |
| EPI_ISL_790733 | Dutch COVID-19 response team | National Institute for Public Health and the Environment (RIVM) | Adam Meijer, Harry Vennema, Dirk Eggink, Jeroen Cremer, Sharon van den Brink, Bas van der Veer, AnneMarie van den Brandt, Florian Zwagemaker, Dennis Schmitz, Chantal Reusken, on behalf of the national COVID-19 response team |
| EPI_ISL_790737 | Dutch COVID-19 response team | National Institute for Public Health and the Environment (RIVM) | Adam Meijer, Harry Vennema, Dirk Eggink, Jeroen Cremer, Sharon van den Brink, Bas van der Veer, AnneMarie van den Brandt, Florian Zwagemaker, Dennis Schmitz, Chantal Reusken, on behalf of the national COVID-19 response team |
| EPI_ISL_790743 | Dutch COVID-19 response team | National Institute for Public Health and the Environment (RIVM) | Adam Meijer, Harry Vennema, Dirk Eggink, Jeroen Cremer, Sharon van den Brink, Bas van der Veer, AnneMarie van den Brandt, Florian Zwagemaker, Dennis Schmitz, Chantal Reusken, on behalf of the national COVID-19 response team |
| EPI_ISL_790784 | Dutch COVID-19 response team | National Institute for Public Health and the Environment (RIVM) | Adam Meijer, Harry Vennema, Dirk Eggink, Jeroen Cremer, Sharon van den Brink, Bas van der Veer, AnneMarie van den Brandt, Florian Zwagemaker, Dennis Schmitz, Chantal Reusken, on behalf of the national COVID-19 response team |
| EPI_ISL_1457743 | Dutch COVID-19 response team | National Institute for Public Health and the Environment (RIVM) | Adam Meijer, Harry Vennema, Dirk Eggink, Jeroen Cremer, Sharon van den Brink, Bas van der Veer, AnneMarie van den Brandt, Florian Zwagemaker, Dennis Schmitz, Chantal Reusken, on behalf of the national COVID-19 response team |
| EPI_ISL_1457752 | Dutch COVID-19 response team | National Institute for Public Health and the Environment (RIVM) | Adam Meijer, Harry Vennema, Dirk Eggink, Jeroen Cremer, Sharon van den Brink, Bas van der Veer, AnneMarie van den Brandt, Florian Zwagemaker, Dennis Schmitz, Chantal Reusken, on behalf of the national COVID-19 response team |
| EPI_ISL_1457753 | Dutch COVID-19 response team | National Institute for Public Health and the Environment (RIVM) | Adam Meijer, Harry Vennema, Dirk Eggink, Jeroen Cremer, Sharon van den Brink, Bas van der Veer, AnneMarie van den Brandt, Florian Zwagemaker, Dennis Schmitz, Chantal Reusken, on behalf of the national COVID-19 response team |
| EPI_ISL_824082 | Dutch COVID-19 response team | National Institute for Public Health and the Environment (RIVM) | Adam Meijer, Harry Vennema, Dirk Eggink, Jeroen Cremer, Sharon van den Brink, Bas van der Veer, AnneMarie van den Brandt, Florian Zwagemaker, Dennis Schmitz, Chantal Reusken, on behalf of the national COVID-19 response team |
| EPI_ISL_824084 | Dutch COVID-19 response team | National Institute for Public Health and the Environment (RIVM) | Adam Meijer, Harry Vennema, Dirk Eggink, Jeroen Cremer, Sharon van den Brink, Bas van der Veer, AnneMarie van den Brandt, Florian Zwagemaker, Dennis Schmitz, Chantal Reusken, on behalf of the national COVID-19 response team |
| EPI_ISL_824087 | Dutch COVID-19 response team | National Institute for Public Health and the Environment (RIVM) | Adam Meijer, Harry Vennema, Dirk Eggink, Jeroen Cremer, Sharon van den Brink, Bas van der Veer, AnneMarie van den Brandt, Florian Zwagemaker, Dennis Schmitz, Chantal Reusken, on behalf of the national COVID-19 response team |
| EPI_ISL_824089 | Dutch COVID-19 response team | National Institute for Public Health and the Environment (RIVM) | Adam Meijer, Harry Vennema, Dirk Eggink, Jeroen Cremer, Sharon van den Brink, Bas van der Veer, AnneMarie van den Brandt, Florian Zwagemaker, Dennis Schmitz, Chantal Reusken, on behalf of the national COVID-19 response team |
| EPI_ISL_824092 | Dutch COVID-19 response team | National Institute for Public Health and the Environment (RIVM) | Adam Meijer, Harry Vennema, Dirk Eggink, Jeroen Cremer, Sharon van den Brink, Bas van der Veer, AnneMarie van den Brandt, Florian Zwagemaker, Dennis Schmitz, Chantal Reusken, on behalf of the national COVID-19 response team |
| EPI_ISL_824137 | Dutch COVID-19 response team | National Institute for Public Health and the Environment (RIVM) | Adam Meijer, Harry Vennema, Dirk Eggink, Jeroen Cremer, Sharon van den Brink, Bas van der Veer, AnneMarie van den Brandt, Florian Zwagemaker, Dennis Schmitz, Chantal Reusken, on behalf of the national COVID-19 response team |
| EPI_ISL_824221 | Dutch COVID-19 response team | National Institute for Public Health and the Environment (RIVM) | Adam Meijer, Harry Vennema, Dirk Eggink, Jeroen Cremer, Sharon van den Brink, Bas van der Veer, AnneMarie van den Brandt, Florian Zwagemaker, Dennis Schmitz, Chantal Reusken, on behalf of the national COVID-19 response team |
| EPI_ISL_905375 | Dutch COVID-19 response team | National Institute for Public Health and the Environment (RIVM) | Adam Meijer, Harry Vennema, Dirk Eggink, Jeroen Cremer, Sharon van den Brink, Bas van der Veer, AnneMarie van den Brandt, Florian Zwagemaker, Dennis Schmitz, Chantal Reusken, on behalf of the national COVID-19 response team |
| EPI_ISL_905377 | Dutch COVID-19 response team | National Institute for Public Health and the Environment (RIVM) | Adam Meijer, Harry Vennema, Dirk Eggink, Jeroen Cremer, Sharon van den Brink, Bas van der Veer, AnneMarie van den Brandt, Florian Zwagemaker, Dennis Schmitz, Chantal Reusken, on behalf of the national COVID-19 response team |
| EPI_ISL_905379 | Dutch COVID-19 response team | National Institute for Public Health and the Environment (RIVM) | Adam Meijer, Harry Vennema, Dirk Eggink, Jeroen Cremer, Sharon van den Brink, Bas van der Veer, AnneMarie van den Brandt, Florian Zwagemaker, Dennis Schmitz, Chantal Reusken, on behalf of the national COVID-19 response team |
| EPI_ISL_905297 | Dutch COVID-19 response team | National Institute for Public Health and the Environment (RIVM) | Adam Meijer, Harry Vennema, Dirk Eggink, Jeroen Cremer, Sharon van den Brink, Bas van der Veer, AnneMarie van den Brandt, Florian Zwagemaker, Dennis Schmitz, Chantal Reusken, on behalf of the national COVID-19 response team |
| EPI_ISL_905298 | Dutch COVID-19 response team | National Institute for Public Health and the Environment (RIVM) | Adam Meijer, Harry Vennema, Dirk Eggink, Jeroen Cremer, Sharon van den Brink, Bas van der Veer, AnneMarie van den Brandt, Florian Zwagemaker, Dennis Schmitz, Chantal Reusken, on behalf of the national COVID-19 response team |
| EPI_ISL_1962985 | Dutch COVID-19 response team | National Institute for Public Health and the Environment (RIVM) | Adam Meijer, Harry Vennema, Dirk Eggink, Jeroen Cremer, Sharon van den Brink, Bas van der Veer, AnneMarie van den Brandt, Florian Zwagemaker, Dennis Schmitz, Chantal Reusken, on behalf of the national COVID-19 response team |
| EPI_ISL_3047866 | Microvida | Microvida | S.D. Pas, J.J. Verweij, J. Stohr |
| EPI_ISL_3047867 | Microvida | Microvida | S.D. Pas, J.J. Verweij, J. Stohr |
| EPI_ISL_2259188 | Microvida | Microvida | S.D. Pas, J.J. Verweij, J. Stohr |
| EPI_ISL_2259136 | Microvida | Microvida | S.D. Pas, J.J. Verweij, J. Stohr |
| EPI_ISL_2259122 | Microvida | Microvida | S.D. Pas, J.J. Verweij, J. Stohr |
| EPI_ISL_1522131 | Dutch COVID-19 response team | National Institute for Public Health and the Environment (RIVM) | Adam Meijer, Harry Vennema, Dirk Eggink, Jeroen Cremer, Sharon van den Brink, Bas van der Veer, AnneMarie van den Brandt, Florian Zwagemaker, Dennis Schmitz, Chantal Reusken, on behalf of the national COVID-19 response team |
| EPI_ISL_1792908 | Dutch COVID-19 response team | National Institute for Public Health and the Environment (RIVM) | Adam Meijer, Harry Vennema, Dirk Eggink, Jeroen Cremer, Sharon van den Brink, Bas van der Veer, AnneMarie van den Brandt, Florian Zwagemaker, Dennis Schmitz, Chantal Reusken, on behalf of the national COVID-19 response team |
| EPI_ISL_1792918 | Dutch COVID-19 response team | National Institute for Public Health and the Environment (RIVM) | Adam Meijer, Harry Vennema, Dirk Eggink, Jeroen Cremer, Sharon van den Brink, Bas van der Veer, AnneMarie van den Brandt, Florian Zwagemaker, Dennis Schmitz, Chantal Reusken, on behalf of the national COVID-19 response team |
| EPI_ISL_1090134 | Dutch COVID-19 response team | National Institute for Public Health and the Environment (RIVM) | Adam Meijer, Harry Vennema, Dirk Eggink, Jeroen Cremer, Sharon van den Brink, Bas van der Veer, AnneMarie van den Brandt, Florian Zwagemaker, Dennis Schmitz, Chantal Reusken, on behalf of the national COVID-19 response team |
